# CHRM4/AKT/MYCN upregulates interferon alpha-17 in the tumor microenvironment to promote neuroendocrine differentiation of prostate cancer

**DOI:** 10.1038/s41419-023-05836-7

**Published:** 2023-05-04

**Authors:** Yu-Ching Wen, Van Thi Ngoc Tram, Wei-Hao Chen, Chien-Hsiu Li, Hsiu-Lien Yeh, Phan Vu Thuy Dung, Kuo-Ching Jiang, Han-Ru Li, Jiaoti Huang, Michael Hsiao, Wei-Yu Chen, Yen-Nien Liu

**Affiliations:** 1 Department of Urology, Wan Fang Hospital, Taipei Medical University, Taipei, 11696 Taiwan; 2 Department of Urology, School of Medicine, College of Medicine, Taipei Medical University, Taipei, 11031 Taiwan; 3 TMU Research Center of Urology and Kidney, Taipei Medical University, Taipei, 11031 Taiwan; 4 International PhD Program in Medicine, College of Medicine, Taipei Medical University, Taipei, 11031 Taiwan; 5 Graduate Institute of Cancer Biology and Drug Discovery, College of Medical Science and Technology, Taipei Medical University, Taipei, 11031 Taiwan; 6 Genomics Research Center, Academia Sinica, Taipei, 11529 Taiwan; 7 Department of Pathology, Duke University Medical Center, Durham, NC 27710 USA; 8 Department of Pathology, Wan Fang Hospital, Taipei Medical University, Taipei, 11696 Taiwan; 9 Department of Pathology, School of Medicine, College of Medicine, Taipei Medical University, Taipei, 11031 Taiwan; 10 TMU Research Center of Cancer Translational Medicine, Taipei Medical University, Taipei, 11031 Taiwan

**Keywords:** Cancer microenvironment, Genetics research, Prognostic markers, Prostatic diseases

## Abstract

Current treatment options for prostate cancer focus on targeting androgen receptor (AR) signaling. Inhibiting effects of AR may activate neuroendocrine differentiation and lineage plasticity pathways, thereby promoting the development of neuroendocrine prostate cancer (NEPC). Understanding the regulatory mechanisms of AR has important clinical implications for this most aggressive type of prostate cancer. Here, we demonstrated the tumor-suppressive role of the AR and found that activated AR could directly bind to the regulatory sequence of muscarinic acetylcholine receptor 4 (*CHRM4*) and downregulate its expression. CHRM4 was highly expressed in prostate cancer cells after androgen-deprivation therapy (ADT). CHRM4 overexpression may drive neuroendocrine differentiation of prostate cancer cells and is associated with immunosuppressive cytokine responses in the tumor microenvironment (TME) of prostate cancer. Mechanistically, CHRM4-driven AKT/MYCN signaling upregulated the interferon alpha 17 (IFNA17) cytokine in the prostate cancer TME after ADT. IFNA17 mediates a feedback mechanism in the TME by activating the CHRM4/AKT/MYCN signaling-driven immune checkpoint pathway and neuroendocrine differentiation of prostate cancer cells. We explored the therapeutic efficacy of targeting CHRM4 as a potential treatment for NEPC and evaluated IFNA17 secretion in the TME as a possible predictive prognostic biomarker for NEPC.

## Introduction

According to an annual report by The American Cancer Society, prostate cancer was predicted to account for 27% of all new cancer cases in American men and almost 11% of cancer-related deaths in 2022 [1]. Androgen deprivation therapy (ADT) is commonly used to reduce the tumor burden in advanced prostate cancer cases [2]. However, long-term anti-androgen receptor (AR) therapy was shown to alter the prostate cell lineage, leading to epithelial-mesenchymal transition (EMT), treatment resistance, and neuroendocrine (NE) differentiation (NED) of prostate cancer cells [2]. NED prostate cancer (NEPC) is an aggressive subtype of advanced prostate cancer. However, NEPC has an unknown pathogenesis, rapidly progresses, responds to low treatment sensitivity, and has an estimated median survival of 10 months from the time of detection [3].

Currently, NEPC cases are mainly treated with ADT combined with abiraterone acetate plus prednisone, docetaxel, enzalutamide/MDV3100, or other anticancer drugs to improve outcomes [4]. However, long-term ADT combination therapy does not significantly improve NEPC patient survival [5]. The mechanism responsible for treatment resistance in NEPC is unclear, the clinical prognosis is poor, and there is a lack of effective diagnostic or prognostic biomarkers. Since ADT treatment may induce prostate cancer cells to undergo NED, detection of gene upregulation after ADT could be used to determine the potential prognosis of NED in prostate cancer patients [5]. We recognize that the discovery of clinically promising NEPC biomarkers is critical to support the diagnosis and development of new strategies for NEPC therapies.

Over the past decade, immunotherapies have shown partially promising results in several cancers, such as leukemia, kidney, and skin cancer, but they face significant challenges when applied to prostate cancer [6]. According to prostate oncologists, the natural position of the prostate is along the urinary tract, a conduit for infectious organisms [7]. Therefore, the prostate may have more immunosuppressive than fighting properties to prevent overreaction against these microorganisms. This may explain why there are few immune cells in the prostate as well as few T-cell signals in prostate tumors [8]. Despite the existence of numerous barriers to T-cell infiltration into prostate cancer cells, the crosstalk between prostate cancer cells and immune cells present in the tumor microenvironment (TME) leading to NED remains unclear [9]. The presence of immune cells surrounding tumor cells, known as tumor-associated macrophages (TAMs), is a major challenge in prostate cancer immunotherapy [10]. TAMs, known as M2-like macrophages, may interact with NEPC-like cells in the TME to support tumor growth and progression [11]. We aimed to investigate the mechanism by which prostate cancer cells interact with immune cells and generate an immunosuppressive TME to promote NEPC progression.

In our previous study, we found that stimulation of the muscarinic acetylcholine receptor 4 (CHRM4)/AKT/MYCN pathway may lead to the development of NEPC in prostate cancer after ADT [12]. We found increased abundances of CHRM4 in high-grade tumors and small-cell prostate cancer (SCPC) samples, suggesting that CHRM4 may serve as a biomarker for predicting advanced prostate cancer. CHRM4 is a G protein-coupled receptor predominantly present in the central nervous system [13], but its role in promoting an immunosuppressive TME has not been precisely characterized. We sought to determine the role of CHRM4 in prostate cancer after ADT and its effect on cytokine responsiveness in the TME for NEPC differentiation.

In this study, we found that ADT resulted in loss of the tumor suppressor effect of AR and reduced binding of AR to the *CHRM4* regulatory sequence, thereby enhancing CHRM4 expression in prostate cancer cells. Abundant CHRM4-driven AKT/MYCN signaling upregulates interferon alpha 17 (IFNA17) cytokine activity in prostate cancer after ADT. Positive correlations between CHRM4 and IFNA17 at the messenger (m)RNA and protein levels, functional characteristics, and clinical datasets were found in advanced and NEPC-like prostate cancers. We demonstrated the abundance of the IFNA17 protein in CHRM4-overexpressing cells and the serum of patients with metastatic prostate cancer, suggesting that IFNA17 is a potential prognostic marker for advanced prostate cancer. Since there are no small molecules or inhibitors known to treat NEPC, we discovered that targeting CHRM4 using Food and Drug Administration (FDA)-approved small compounds may inhibit tumor growth and NED in prostate cancer cells in vitro and in vivo.

## Results

### AR suppression induces CHRM4 expression in prostate cancer

**Fig. 1:**
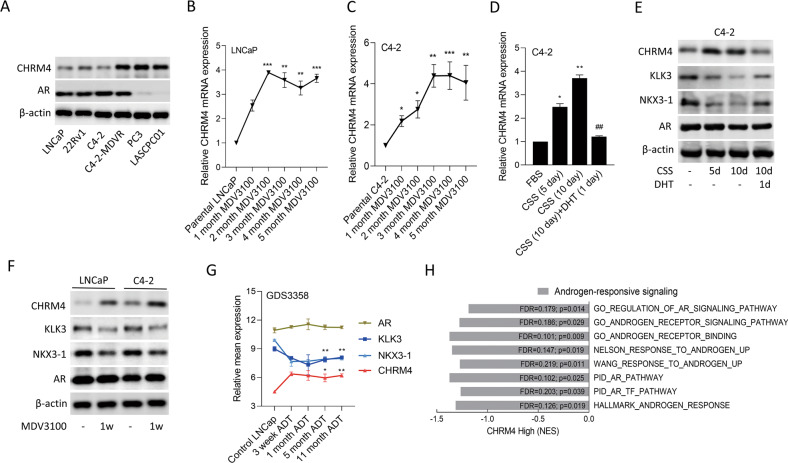
Upregulation of CHRM4 is associated with prolonged androgen withdrawal. **A** CHRM4 and AR protein levels of LNCaP, 22Rv1, C4-2, C4-2-MDVR, PC3, and LASCPC01 cells, measured by a western blot analysis. **B**, **C** CHRM4 mRNA abundances in LNCaP and C4-2 cells during 1, 2, 3, 4, and 5 months of 20 μM MDV3100 treatment, measured by an RT-qPCR analysis. * vs. parental LNCaP or C4-2 cells, by a one-way ANOVA. **D** Relative CHRM4 mRNA levels of C4-2 cells cultured in charcoal-stripped serum (CSS)-containing medium for 5 and 10 days, followed by treatment with 10 nM dihydrotestosterone (DHT) for 24 h. Quantification of relative mRNA levels is presented as the mean ± SEM of three biological replicates. *p < 0.05, **p < 0.01, ***p < 0.0001. **E** CHRM4, androgen-responsive markers (KLK3 and NKX3-1), and AR protein levels in C4-2 cells cultured in CSS-containing medium for 5 and 10 days, followed by treatment with 10 nM DHT for 24 h. **F** CHRM4, KLK3, NKX3-1, and AR protein levels of LNCaP and C4-2 cells cultured in 20 μM MDV3100 for 1 week. **G** Relative mean expressions of the AR, KLK3, NKX3-1, and CHRM4 in LNCaP cells from 3 weeks to 11 months of androgen withdrawal (ADT) in the GDS3358 database. * vs. the control, by a one-way ANOVA. **H** GSEAs of TCGA prostate dataset revealing negative associations between high CHRM4 expression in prostate tissues with gene signatures representing androgen-responsive signaling (GO, Nelson, Wang, PID, and Hallmark). NES normalized enrichment score, FDR false discovery rate.

To understand expression patterns of CHRM4 in relation to AR inhibition in prostate cancer, we examined CHRM4 and AR protein expression levels in androgen-dependent prostate cancer cells (LNCaP), CRPC cells (22Rv1 and C4-2), CRPC cells after long periods of AR antagonist/MDV3100 treatment (C4-2-MDVR), AR-negative prostate cancer cells (PC3), and NEPC-like cells (LASCPC01). We found that the C4-2-MDVR, PC3, and LASCPC01 cell lines exhibited relatively higher expression levels of CHRM4 compared to androgen-sensitive LNCaP cells (Fig. 1A). In contrast, relatively low or negative AR expression levels were found in PC3 and LASCPC01 cells (Fig. 1A), suggesting that AR expression may be involved in regulating CHRM4 abundance in PC3 and LASCPC01 cells. However, C4-2-MDVR cells expressed high levels of the AR regardless of high CHRM4 expression. When LNCaP and C4-2 cells were treated with MDV3100 for 1 to 5 months, results demonstrated that MDV3100-treated cells had significantly increased CHRM4 mRNA levels compared to untreated cells (Fig. 1B, C). In line with AR inhibition, C4-2 cells were cultured in charcoal-stripped serum (CSS)-containing medium to mimic ADT, which increased CHRM4 mRNA expression levels at 5 and 10 d; however, treatment with the AR-ligand, dihydrotestosterone (DHT), abolished these effects, resulting in a significant decrease in CHRM4 mRNA levels (Fig. 1D). We also found that increased levels of the CHRM4 protein in cells were associated with decreased KLK3 and NKX3-1 proteins, which are AR response markers, following treatment with CSS-containing medium, whereas DHT treatment reversed these protein levels, but did not affect AR expression (Fig. 1E). We also found that when LNCaP and C4-2 cells were treated with the AR antagonist, MDV3100, CHRM4 protein levels significantly increased, but KLK3 and NKX3-1 protein levels decreased compared to those in control cells (Fig. 1F). Moreover, AR expression did not significantly differ among AR antagonist treatments. We hypothesized that AR inhibition might prevent the nuclear translation of the AR and binding of the AR to downstream targets. To confirm the effects of ADT on the expression of these genes, we examined mean expression levels of *CHRM4*, *KLK3*, *NKX3-1*, and *AR* mRNAs from the GDS3358 database. Results showed that an increase in CHRM4 was significantly associated with reductions in KLK3 and NKX3-1 in LNCaP cells cultured for 3 weeks to 11 months, but no change in the AR was found (Fig. 1G), which is consistent with our results. Moreover, tissues expressing high CHRM4 levels were significantly correlated with downregulated AR-responsive gene signatures (GO, Wang [13], Nelson [14], PID, and Hallmark), as revealed by a gene set enrichment analysis (GSEA) in The Cancer Genome Atlas (TCGA) prostate cancer dataset (Fig. 1H). These data suggest that prolonged inhibition of AR signaling may result in the downregulation of AR-responsive markers and upregulation of CHRM4 in prostate cancer cells.

### Androgen-activated AR downregulates CHRM4 expression

**Fig. 2:**
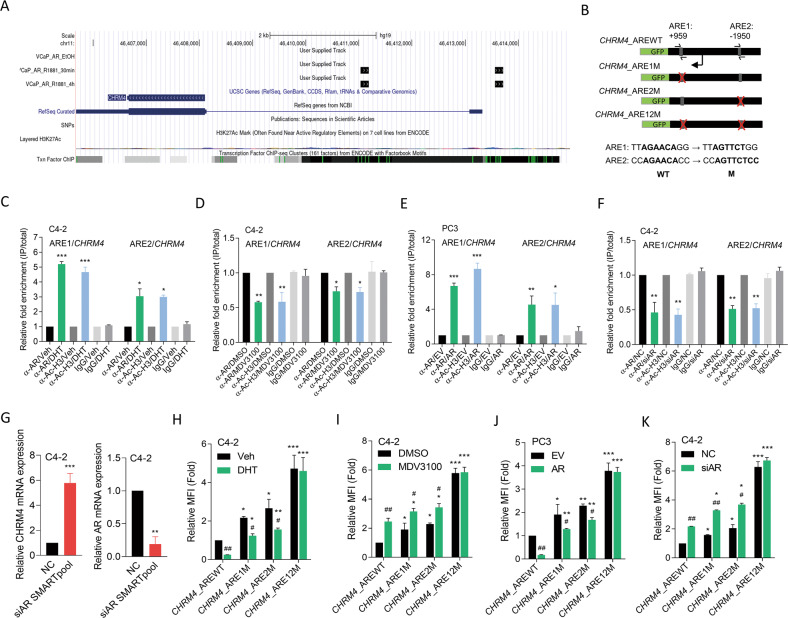
*CHRM4* is downregulated by the AR in prostate cancer cells. **A** ChIP-sequencing analysis of the detected DNA-binding sites for the AR of the *CHRM4* gene in cells in response to 0.5 or 4 h of AR-ligand R1881 treatment labeled as black boxes in the tracks. ChIP-sequencing data were downloaded from the Gene Expression Omnibus (GEO) (GSE84432) and analyzed by Genome Brower (Genomics Institute). **B** Schematic of the predicted AR resonse elements (AREs) and an introduced single- or double-binding site mutant in regulatory sequence reporter constructs of human *CHRM4* (GRCh38:11). ChIP assay showing binding of the AR and acetyl-H3 to predicted AREs of the *CHRM4* gene regulatory sequence following treatment of C4-2 cells with 10 nM dihydrotestosterone (DHT) (**C**) or 20 μM MDV3100 (**D**) for 24 h. Sheared chromatin from nuclear extracts was precipitated with antibodies to the AR and acetyl-H3, and predictive primers (**B**, black arrows) were used to quantify the precipitated DNA by a qPCR. Enrichment of each protein to each site is given as a percentage of the total input and then normalized to IgG. * vs. the vehicle (Veh) (**C**) or DMSO (**D**), by a one-way ANOVA. **E**, **F** ChIP assay showing binding of the AR and acetyl-H3 to predicted AREs of the *CHRM4* gene regulatory sequence in PC3 cells following stable transfection with an empty vector (EV) or AR cDNA vector (**E**) or in C4-2 cells with a non-targeting control (NC) or AR siRNA transfection (**F**). * vs. the EV (**E**) or NC (**F**), by a one-way ANOVA. **G** Relative CHRM4 and AR mRNA levels of C4-2 cells transfected with the NC or AR siRNA, measured by an RT-qPCR analysis, * vs. the NC. **H**, **I** Relative mean florescence intensity (MFI) of the *GFP* reporter gene containing a wild-type (WT)- or mutant (M)-ARE from the *CHRM4* regulatory sequence in C4-2 cells following treatment with 10 nM DHT (**H**) or 20 μM MDV3100 (**I**) for 48 h. * vs. WT; ^#^ vs. the vehicle (Veh) (**H**) or DMSO (**I**), by a two-way ANOVA. **J**, **K** Relative MFI of the *GFP* reporter gene containing a WT- or M-ARE from the *CHRM4* regulatory sequence in PC3 cells following stable transfection with the EV or AR cDNA vector (**J**) or in C4-2 cells following NC or AR siRNA transfection (**K**). * vs. the EV (**J**) or NC (**K**), by a two-way ANOVA. Quantification of the ChIP assay, relative MFI values, and mRNA levels are presented as the mean ± SEM from three biological replicates. *p < 0.05, **p < 0.01, ***p < 0.001.

The AR is a key factor in the differentiation of luminal epithelial cells and was shown to play a tumor-suppressive role in breast and prostate cancers [15, 16]. Therefore, its inhibitory effect may activate a carcinogenic pathway. We hypothesized that the AR acts as a transcriptional repressor of *CHRM4*. We downloaded chromatin immunoprecipitation (ChIP)-sequencing data from the Gene Expression Omnibus (GEO) (GSE84432) and analyzed it using the Genome Brower (Genomics Institute, UCSC, CA, USA). Results revealed that the AR appeared to bind to multiple sites of the *CHRM4* gene in VCaP cells after AR-ligand treatment (Fig. 2A). We searched for sequences resembling the AR response element (ARE) [17] in *CHRM4* regulatory sequences. We found one putative ARE upstream and one downstream of the *CHRM4* transcription start site (Fig. 2B). Following DHT or MDV3100 treatment, ChIP assays were performed using an antibody against the AR and a positive control anti-acetyl-histone H3 antibody in C4-2 cells to determine the regulatory mechanisms by which AR gain- or loss-of-function directly interacts with *CHRM4*. We found that the AR-binding capacity of ARE1 and ARE2 on *CHRM4* significantly increased after DHT treatment but decreased in MDV3100-treated cells (Fig. 2C, D). In addition, AR overexpression in PC3 cells increased the binding ability of the AR to ARE1 and ARE2 (Fig. 2E). However, AR-knockdown (KD) in C4-2 cells reduced the binding ability of the AR to ARE1 and ARE2 (Fig. 2F). We also found that CHRM4 mRNA levels significantly increased in C4-2 cells with AR-KD compared to those in control cells (Fig. 2G), suggesting a negative interaction between CHRM4 and AR expression. Reporter assays were performed using a DNA construct containing wild-type (WT) and mutant (M) ARE1 and ARE2 on the *CHRM4* regulatory sequence cloned into a green fluorescent protein (GFP) reporter (Fig. 2B). DHT-treated C4-2 cells showed significantly decreased WT reporter gene activity compared to untreated cells, whereas cells treated with MDV3100 showed increased WT reporter gene activity (Fig. 2H, I). Despite a single mutation of ARE1 or ARE2 showing a significant reduction or induction of reporter activity compared to the WT ARE, a double mutation abolished the effect of DHT or MDV3100 on reporter gene activity, respectively, compared to vehicle or DMSO treatment (Fig. 2H, I). Moreover, AR overexpression in PC3 cells downregulated WT reporter activity compared to empty vector (EV)-expressing cells, whereas C4-2 cells with AR-KD upregulated WT reporter activity compared to non-targeting control (NC)-expressing cells (Fig. 2J, K). ARE double mutants abolished reporter activity affected by AR overexpression or KD (Fig. 2J, K), supporting both ARE sites having an efficient AR-binding capacity to downregulate *CHRM4*. These results suggest that AR inhibition may upregulate CHRM4 expression, supporting our previous finding that ADT may induce an abundance of CHRM4 [12].

### CHRM4 overexpression is associated with NED in prostate cancer cells

**Fig. 3:**
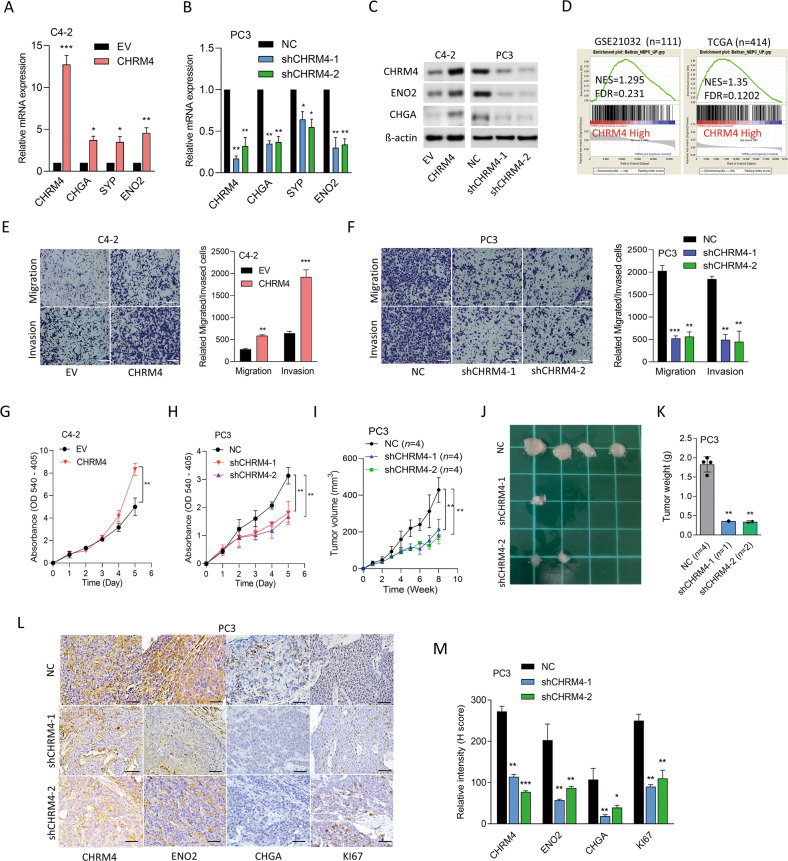
Increased CHRM4 is associated with oncogenic features and neuroendocrine differentiation in prostate cancer. **A**, **B** CHRM4 and NE marker (CHGA, SYP, and ENO2) mRNA levels in C4-2 cells stably transfected with an empty vector (EV) or a CHRM4-expressing vector (**A**) or in PC3 cells stably transfected with the non-targeting control (NC) or CHRM4 shRNA vector **(B)**, measured by an RT-qPCR analysis, * vs. the EV (**A**) or NC (**B**), by a one-way ANOVA. **C** Western blot showing CHRM4 and NE marker protein levels in CHRM4-modified C4-2 and PC3 cells. **D** GSEA analysis of TCGA prostate cancer dataset revealed positive correlations between higher CHRM4 expression in prostate tissues and gene profiles reflecting NEPC-responsiveness. NES, normalized enrichment score; FDR, false discovery rate. **E**–**H** Relative cell migration and invasion through Matrigel **(E**, **F)** and proliferation **(G**, **H)** of CHRM4-overexpressing C4-2 (**E**, **G**) or CHRM4-knockdown (KD) PC3 cells (**F**, **H**). *n* = 5 per group. * vs. the EV (**E**, **G**) or NC (**F**, **H**), by a one-way ANOVA. **I**–**K** Tumor growth analysis (**I**, **J**) and tumor weights (**K**) of CHRM4-KD PC3 cells subcutaneously inoculated into male nude mice for 8 weeks. Tumor weights were measured on the day tumors were collected. Tumor sizes were measured once a week and analyzed by a one-way ANOVA. IHC staining (**L**) and representative intensities (**M**) of CHRM4, ENO2, CHGA, and KI67 in subcutaneous tumors from **J**. * vs. NC-bearing tumors, by a two-tailed Student’s *t*-test. Quantification of relative mRNA levels, and migration, invasion, and proliferation are presented as the mean ± SEM from three biological replicates. *p < 0.05, **p < 0.01, ***p < 0.001.

To investigate the role of CHRM4 in NED progression in prostate cancer, CHRM4 was overexpressed or knocked-down in AR-positive C4-2 and AR-negative PC3 cells, respectively. CHRM4 overexpression increased mRNA and protein levels of NE markers in C4-2 cells compared to cells transfected with the EV (Fig. 3A, C). In contrast, PC3 cells with CHRM4-KD exhibited significant decreases in mRNA and protein levels of NE markers compared to those in control cells (Fig. 3B, C). We also found that CHRM4-KD in the LASCPC01 NEPC cell line decreased the abundance of NE markers (Supplementary Fig. S1A, B). Additionally, in an analysis of prostate cancer datasets (GSE21032 and TCGA), CHRM4 overexpression was positively correlated with an NEPC-response gene signature (Fig. 3D). Next, we assessed the role of CHRM4 in prostate cancer cells and found that CHRM4-overexpressing C4-2 cells exhibited increased rates of cell migration and invasion through Matrigel (Fig. 3E). Conversely, these effects were reduced in PC3 cells with CHRM4-KD (Fig. 3F). We further evaluated the relevance of CHRM4-mediated proliferation in both C4-2 and PC3 cells. CHRM4 overexpression resulted in upregulated proliferation compared to EV-expressing cells (Fig. 3G), whereas cells with CHRM4-KD showed downregulation of the proliferation rate compared to NC-expressing cells (Fig. 3H). When mice were subcutaneously injected with PC3 cells with CHRM4-KD, we observed that both tumor size and weight significantly decreased in CHRM4-KD cell-bearing mice relative to control cell-bearing mice (Fig. 3I–K). We also found reduced abundances of CHRM4, NE markers (ENO2 and CHGA), and a proliferation marker (Ki67) in tumors of mice bearing CHRM4-KD PC3 cells relative to mice bearing control cells by immunohistochemical (IHC) staining (Fig. 3L, M). These findings suggest that CHRM4 inhibition may reduce expressions of NE markers, tumor growth, and functional characteristics of malignant progression, and may contribute to the development of NED in prostate cancer.

### Abundant CHRM4 correlates with the IFNA17 cytokine response in prostate cancer

**Fig. 4:**
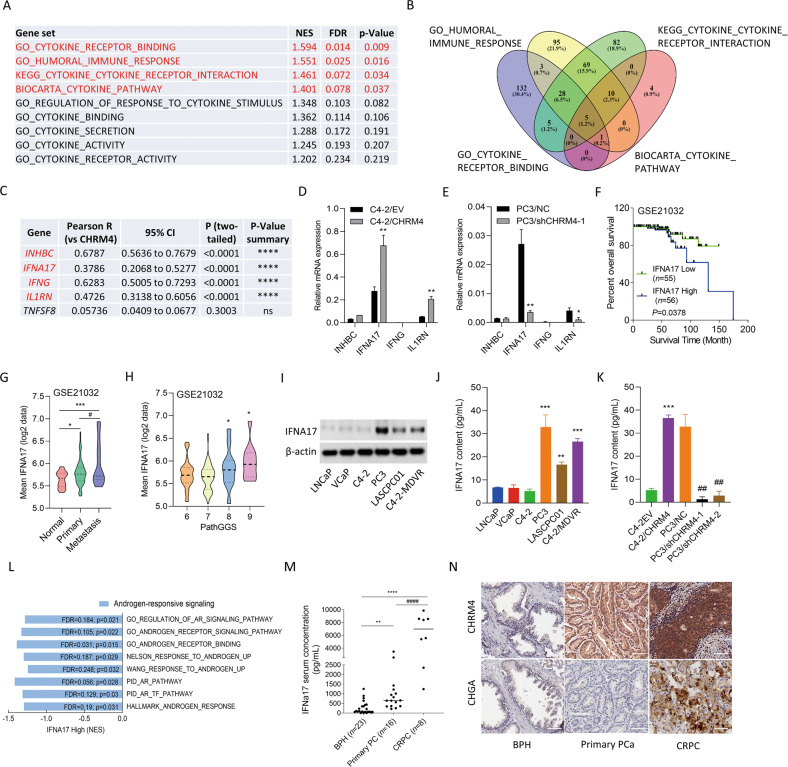
CHRM4 is correlated with IFNA17 responsiveness in prostate cancer. **A** GSEAs of TCCA prostate cancer dataset showing that high abundance of CHRM4 mRNA in prostate cancer samples was positively linked to a wide range of cytokine-responsive gene signatures (GO, KEGG, and BIOCARTA). NES, normalized enrichment score; FDR, false discovery rate. **B** Venn diagram showing the number of overlapping genes identified in the top four cytokine-responsive gene sets. **C** The list of five overlapping gene candidates includes *INHBC, IFNA17, IFNG, IL1RN*, and *TNFSF8* from (**B**). Pearson correlations among the five candidate genes and *CHRM4* were analyzed in TCGA prostate cancer dataset by XY correlation analyses in GraphPad Prism. Relative mRNA levels of *INHBC, IFNA17, IFNG*, and *IL1RN* in C4-2 cells stably transfected with an empty vector (EV) or a CHRM4-expressing vector (**D**) or in PC3 cells stably transfected with a non-targeting control (NC) or CHRM4 shRNA vector, examined by an RT-qPCR. * vs. the EV (**D**) or NC (**E**), by a one-way ANOVA. **F** Kaplan–Meier analyses of IFNA17 alterations in the GSE21032 dataset. A log-rank (Mantel–Cox) test was used for the survival curve analysis. Hazard ratio = 0.3806, *p* = 0.0378. **G** Mean expression levels of IFNA17 in normal prostate (*n* = 28), primary prostate cancer (*n* = 111), and metastatic prostate cancer (*n* = 13) samples in the GSE21032 dataset. * vs. normal prostate; ^#^ vs. Primary, *p < 0.05, ***p < 0.001, by a two-way ANOVA. **H** Mean expression levels of IFNA17 in prostate cancer patient samples in the GSE21032 dataset by pathologic Gleason scores (GSs). * vs. GS6. * p < 0.05, by a one-way ANOVA. **I** IFNA17 protein levels in LNCaP, VCaP, C4-2, PC3, LASCPC01, and C4-2-MDVR cells, measured by a Western blot analysis. IFNA17 cytokine concentrations in supernatants of cultured medium derived from LNCaP, VCaP, C4-2, PC3, LASCPC01, and C4-2-MDVR cells (**J**) or C4-2 and PC3 cells expressing the EV and CHRM4 cDNA or the NC and CHRM4 shRNA vectors (**K**), measured with an ELISA kit. * vs. LNCaP cells (**J**) or the EV (**K**); ^#^ vs. the NC (**K**), by a two-way ANOVA. Quantification of relative mRNA levels and IFNA17 contents is presented as the mean ± SEM from three biological replicates. *p < 0.05, **p < 0.01, ***p < 0.001. **L** GSEAs of TCGA prostate dataset revealing negative associations between high IFNA17 expression in prostate tissues with gene signatures representing androgen-responsive signaling (GO, Nelson, Wang, PID, and Hallmark). NES normalized enrichment score, FDR false discovery rate. **M** IFNA17 cytokine concentrations in patient sera derived from benign prostatic hyperplasia (BHP; *n* = 23), primary prostate cancer (*n* = 16), and castration-resistant prostate cancer (CRPC) samples (*n* = 8). * vs. BPH; ^#^ vs. primary prostate cancer, by a two-way ANOVA. **N** Representative images of IHC staining of CHRM4 and CHGA in selected tissue sections from patients diagnosed with BHP, primary prostate cancer, and CRPC from (**M**).

To understand how CHRM4 expression is involved in microenvironmental variables, we examined the association between CHRM4 and cytokine responsiveness in prostate cancer using TCGA prostate cancer datasets. We found that tissues expressing high levels of CHRM4 were positively associated with gene signatures for cytokine responsiveness, and we focused on GSEA-based normalized enrichment scores (NESs) with adjusted thresholds of ≥1.4 for the top four gene signatures (Fig. 4A). Based on the GSEA positive rank metric score, correlations between cytokine response signature components and CHRM4 expression were examined to verify the positive association between the cytokine response and CHRM4 upregulation. A Venn diagram revealed that the *INHBC, IFNA17, IFNG, IL1RN*, and *TNFSF8* genes overlapped among the top four cytokine-responsive signatures (Fig. 4B, C). These five candidate genes were validated by a Pearson correlation analysis, and the *INHBC, IFNA17, IFNG*, and *IL1RN* genes positively correlated with CHRM4 were collected according to the significance of confidence intervals and *p* values (*p* < 0.0001, Fig. 4C). Four candidate genes were validated by measuring their mRNA levels in CHRM4-expressing C4-2 and CHRM4-KD PC3 cells. Consequently, CHRM4 overexpression significantly increased mRNA levels of IFNA17 and IL1RN in C4-2 cells compared to those in control cells (Fig. 4D). In contrast, PC3 cells with CHRM4-KD exhibited decreased IFNA17 and IL1RN mRNA levels (Fig. 4E). Based on their expression levels, we selected the most altered candidate gene, *IFNA17*, for further study. Kaplan-Meier survival analysis revealed that samples with greater IFNA17 expression had lower survival rates in the GSE21032 dataset (Fig. 4F). We validated the association between IFNA17 levels and tumor grade and confirmed that IFNA17 was enhanced in primary and metastatic prostate cancer samples compared to those in normal prostate (Fig. 4G) and in prostate cancer samples with high Gleason scores (Fig. 4H). Next, we determined IFNA17 protein levels in various prostate cancer cell lines. Consistent with CHRM4 expression (Fig. 1A), IFNA17 protein expression was significantly higher in AR-negative PC3, NEPC-like LASCPC01, and MDV3100-resistant C4-2-MDVR cells than in androgen-dependent LNCaP cells (Fig. 4I). Consistently, PC3, LASCPC01, and C4-2-MDVR cell culture supernatants contained significantly elevated IFNA17 cytokine levels compared to LNCaP cell culture supernatants as analyzed by an enzyme-linked immunosorbent assay (ELISA) (Fig. 4J). Abundant serum IFNA17 levels were also found in CHRM4-expressing C4-2 cells, whereas PC3 cells with CHRM4-KD showed reduced serum IFNA17 levels (Fig. 4K). In addition, tissues expressing high IFNA17 levels were negatively correlated with AR-responsive gene signatures, as revealed by GSEAs in TCGA prostate cancer datasets (GO, Nelson, Wang, PID; Fig. 4L). To determine the clinical relevance, we examined IFNA17 cytokine concentration released by prostate cancer patients using sera collected from the Taipei Medical University-Wan Fang Hospital (Taipei, Taiwan). Results showed that the IFNA17 cytokine was upregulated in primary prostate cancer samples compared to the benign prostatic hyperplasia (BPH) group, and higher levels of IFNA17 were found in CRPC patients than in the BPH or primary prostate cancer groups (Fig. 4M). Furthermore, IHC staining showed higher intensity for CHRM4 and an NE marker (CHGA) in selected CRPC patient samples than in BPH or primary patient groups (Fig. 4N). In summary, IFNA17 cytokine secretion may be positively correlated with CHRM4 abundance, and may be involved in NED progression in advanced prostate cancer.

### IFNA17-driven NED and malignancy are associated with immune checkpoint signaling

**Fig. 5:**
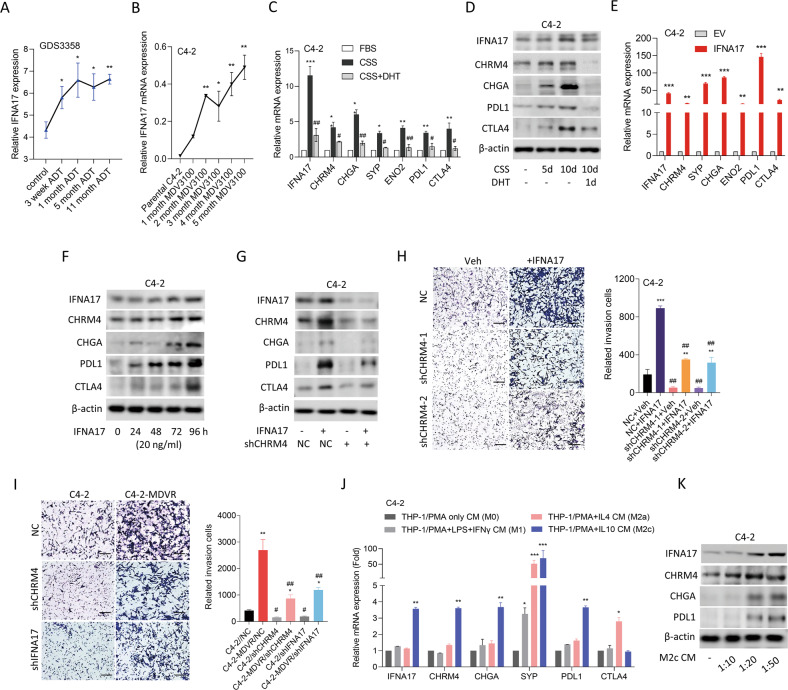
Increased IFNA17 is correlated with neuroendocrine differentiation of prostate cancer after androgen-deprivation therapy. **A** Mean expression levels of IFNA17 in LNCaP cells from the GDS3358 database during 3~11 months of ADT. * vs. the control, *p < 0.05, **p < 0.01, by a one-way ANOVA. **B** Relative IFNA17 mRNA abundances in C4-2 cells following 1~5 months of 20 μM MDV3100 treatment relative to parental C4-2 cells, measured with an RT-qPCR analysis. * vs. parental C4-2 cells, by a one-way ANOVA. **C** Relative mRNA levels of IFNA17, CHRM4, NE markers (CHGA, SYP, and ENO2), and immune checkpoints (PDL1 and CTLA4) in C4-2 cells cultured in charcoal-stripped serum (CSS)-containing medium for 5 days, followed by treatment with 10 nM dihydrotestosterone (DHT) for 24 h. **D** Immunoblots showing IFNA17, CHRM4, CHGA, PDL1, and CTLA4 protein levels in C4-2 cells cultured in CSS-containing medium for 5 and 10 days, followed by treatment with 10 nM DHT for 24 h after 10 days. **E** Relative IFNA17, CHRM4, NE marker, and immune checkpoint mRNA levels in C4-2 cells stably transfected with an empty vector (EV) or IFNA17-expressing vector, measured by an RT-qPCR analysis. * vs. the EV, by a one-way ANOVA. **F** Immunoblots of IFNA17, CHRM4, CHGA, PDL1, and CTLA4 in C4-2 cells exposed to 20 ng/mL IFNA17 protein at different time points. **G** Immunoblots showing IFNA17, CHRM4, CHGA, PDL1, and CTLA4 protein levels in C4-2 cells expressing a non-targeting control (NC) or CHRM4 shRNA following 20 ng/mL IFNA17 protein treatment for 48 h. **H** Relative invasion through Matrigel of C4-2 cells expressing the NC or CHRM4 shRNA following 20 ng/mL IFNA17 protein treatment for 24 h. *n* = 5 per group. * vs. the vehicle (Veh); ^#^ vs. the NC, by a two^-^way ANOVA. **I** Relative invasion through Matrigel in C4-2 and C4-2-MDVR cells stably transfected with the NC, IFNA17, or CHRM4 shRNA for 12 h. *n* = 5 per group. * vs. parental C4-2; ^#^ vs. the NC, by a two^-^way ANOVA. **J** Relative mRNA levels of IFNA17, CHRM4, NE markers, and immune checkpoints in C4-2 cells treated with conditioned medium (CM) of THP-1 cells treated with PMA or following with lipopolysaccharide (LPS) + IFN-γ, IL-4, or IL-10 cytokine treatment for 48 h. * vs. PMA only CM. **K** Immunoblots showing IFNA17, CHRM4, CHGA, and PDL1 protein levels in C4-2 cells treated with various concentrations of CM collected from M2c-like macrophages for 48 h. Quantification of relative mRNA levels and cell invasion through Matrigel is presented as the mean ± SEM from three biological replicates. *p < 0.05, **p < 0.01, ***p < 0.001.

ADT was shown to be the main factor driving NED in prostate cancer [2]. To determine the influence of ADT on *IFNA17* expression, we investigated mean mRNA expression levels in LNCaP cells cultured with ADT for 11 months. Results showed that IFNA17 levels significantly increased after 3 weeks of ADT (Fig. 5A). Consistently, elevated IFNA17 mRNA levels were found in C4-2 cells cultured with MDV3100 for 2~5 months compared to parental C4-2 cells (Fig. 5B). Interestingly, mimic AR inhibition in cells cultured in CSS-containing medium not only exhibited increased abundance of CHRM4 and IFNA17, but also increased mRNA and protein expression of NE markers and immune checkpoints (PDL1 and CTLA4); however, these changes were suppressed in cells treated with additional DHT, resulting in a decrease in their mRNA and protein levels (Fig. 5C, D). These data suggest a positive correlation between CHRM4 and IFNA17 following AR inhibition, which may be associated with immune checkpoint responses in the TME. Moreover, overexpression of IFNA17 complementary (c)DNA in C4-2 cells substantially increased mRNA levels of NE markers and immune checkpoints compared to EV-expressing cells (Fig. 5E). IFNA17 protein-treated C4-2 cells showed a time-dependent increase in protein levels of CHRM4 associated with induction of NE marker and immune checkpoint compared to untreated cells (Fig. 5F). However, C4-2 cells with CHRM4-KD showed reduced IFNA17-driven effects on CHRM4, NE marker, and immune checkpoint abundance (Fig. 5G), suggesting that IFNA17-driven stimulation of NE markers and immune checkpoint signaling occurs in a CHRM4-dependent manner. IFNA17 overexpression in C4-2 cells resulted in increased relative cell invasion through Matrigel compared to EV-expressing cells, whereas CHRM4-KD in cells inhibited the effect of the IFNA17 protein on increasing cell invasion (Fig. 5H). MDV3100-resistant C4-2 cells promoted relative cell invasion compared to control cells; however, MDV3100-resistant C4-2 cells with IFNA17-KD or CHRM4-KD showed significantly reduced relative cell invasion through Matrigel compared to NC-expressing cells (Fig. 5I). To investigate the mechanisms by which prostate cancer cells interact with M2-like macrophages in the TME to drive NED and immunosuppressive responses, the human THP-1 monocytic cell line was cultured in phorbol 12-myristate 13-acetate (PMA) to induce macrophage-like cell differentiation. PMA-treated THP-1 cells acquired M2-like macrophages following interleukin (IL)-4 or IL-10 treatment. We also examined M1-like macrophages in PMA-treated THP-1 cells treated with interferon (IFN)-γ. We found that conditioned medium (CM) collected from M2-like (M2c) macrophages cocultured with C4-2 cells had enhanced IFNA17 and CHRM4 expressions associated with NE marker (CHGA and SYP) and immune checkpoint (PDL1) abundances compared to PMA only or M1-like macrophage-CM treatment (Fig. 5J). Interestingly, M2c macrophages-CM treatment in C4-2 cells revealed enhanced IFNA17 and CHRM4 protein levels, which were correlated with increased CHGA and PDL1 protein abundances in a dose-dependent manner (Fig. 5K). These results suggest that interactions between prostate cancer cells and M2c macrophages may drive the immunosuppressive TME, where increased IFNA17 and CHRM4 levels may promote NEPC differentiation and immune checkpoint abundance in prostate cancer cells.

### IFNA17 is regulated by ADT-induced CHRM4/AKT/MYCN signaling

**Fig. 6:**
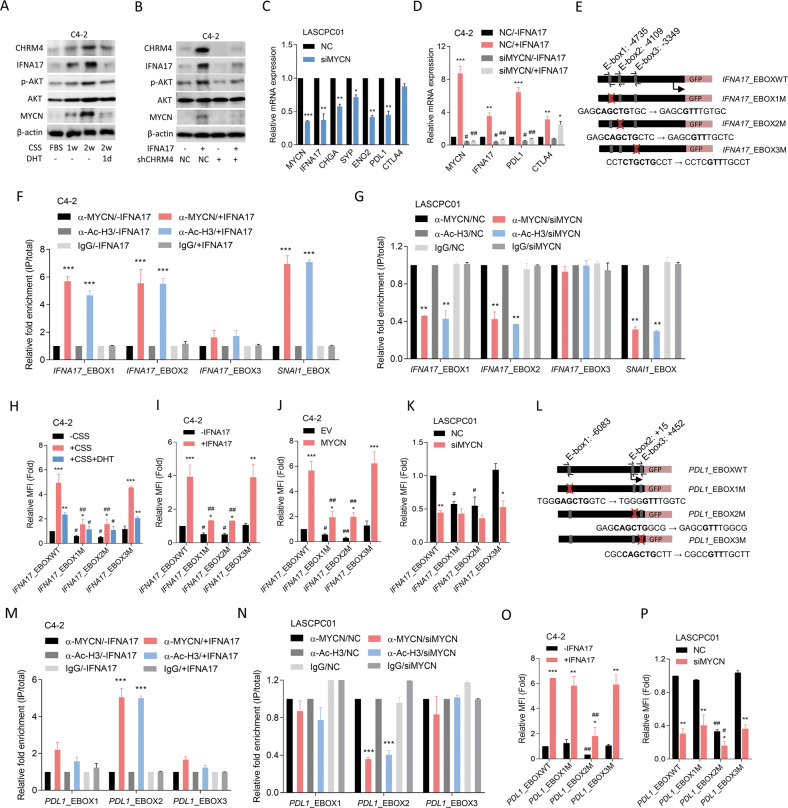
CHRM4/AKT/MYCN upregulates *IFNA17* and *PDL1* in prostate cancer after androgen-deprivation therapy. **A** Immunoblots of CHRM4, IFNA17, phosphorylated (p)-AKT, AKT, and MYCN proteins in C4-2 cells cultured in charcoal-stripped serum (CSS)-containing medium for 1 or 2 weeks, followed by treatment with 10 nM dihydrotestosterone (DHT) for 24 h. **B** Immunoblots showing CHRM4, IFNA17, p-AKT, AKT, and MYCN protein levels in C4-2 cells expressing a non-targeting control (NC) or CHRM4 shRNA vector, then treated with 20 ng/ml IFNA17 protein for 24 h. **C** Relative mRNA levels of MYCN, IFNA17, neuroendocrine (NE) markers (CHGA, SYP, and ENO2), and immune checkpoints (PDL1 and CTLA4) in LASCPC01 cells with NC or MYCN siRNA transfection, measured by an RT-qPCR analysis. * vs. the NC, by a one-way ANOVA. **D** Relative mRNA levels of MYCN, IFNA17, PDL1, and CTLA4 in C4-2 cells with NC or MYCN siRNA transfection, followed 20 ng/ml IFNA17 protein treatment for 24 h, as measured by an RT-qPCR analysis. * vs. -IFNA17; ^#^ vs. the NC, by a two-way ANOVA. **E** Schematic of the predicted E-boxes and an introduced single-binding site mutant in regulatory sequence reporter constructs of human *IFNA17* (GRCh38:9). **F** ChIP assay showing binding of MYCN and acetyl-H3 to predicted E-box1 and E-box2 of the *IFNA17* gene regulatory sequence in C4-2 cells following treatment with 20 ng/ml of the IFNA17 protein for 24 h. Sheared chromatin from nuclear extracts was precipitated with antibodies to MYCN and acetyl-H3, and predictive primers (**E**, black arrows) were used to quantify the precipitated DNA by a qPCR. Enrichment of each protein to each site is given as a percentage of the total input and then normalized to IgG. * vs. -IFNA17, by a one-way ANOVA. **G** ChIP assay showing reduced binding of MYCN and acetyl-H3 to the predicted E-box1 and E-box2 of the *IFNA17* gene regulatory sequence in LASCPC01 cells with NC or MYCN siRNA transfection. * vs. the NC, by a one-way ANOVA. Relative mean florecence intesity (MFI) of the GFP reporter gene containing wild-type (WT)- or mutant (M)-E-boxes from the *IFNA17* regulatory sequence in C4-2 cells following CSS-containing medium or 10 nM DHT (**H**) or 20 ng/ml IFNA17 protein (**I**) treatment for 48 h. * vs. -CSS (**H**) or -IFNA17 (**I**); ^#^ vs. the WT, by a two-way ANOVA. Relative MFI of the GFP reporter gene containing WT- or M-E-boxes from the *IFNA17* regulatory sequence in C4-2 cells co-transfected with the EV or MYCN cDNA vector (**J**), or NC or MYCN siRNA (**K**) for 48 h. * vs. the EV (**J**) or the NC (**K**); ^#^ vs. the WT, by a two-way ANOVA. **L** Schematic of the predicted E-boxes and an introduced single-binding site mutant in regulatory sequence reporter constructs of human *PDL1* (GRCh38:9). ChIP assay showing increased binding of MYCN and acetyl-H3 to predicted E-box2 of *PDL1* gene regulatory sequence in C4-2 cells with 20 ng/ml IFNA17 protein treatment for 48 h (**M**) or in LASCPC01 cells with NC or MYCN siRNA transfection (**N**). * vs. -IFNA17 (**M**) or the NC (**N**), by a one-way ANOVA. Relative MFI of the GFP reporter gene containing WT- or M-E-boxes from the *PDL1* regulatory sequence in C4-2 cells with 20 ng/ml IFNA17 protein treatment for 48 h (**O**) or in LASCPC01 cells with NC or MYCN siRNA transfection (**P**). * vs. -IFNA17 (**O**) or the NC (**P**); ^#^ vs. the WT, by a two-way ANOVA. Quantification of the ChIP assay and relative MFI values are presented as the mean ± SEM from three biological replicates. *p < 0.05, **p < 0.01, ***p < 0.001.

Our previous study showed that an increase in CHRM4 protein levels after ADT is involved in the activation of AKT/MYCN signaling [12]. To assess whether IFNA17 upregulation is associated with CHRM4/AKT/MYCN signaling after ADT, AR-positive C4-2 cells were cultured in CSS-containing medium. We found that increased IFNA17 protein expression was associated with induction of CHRM4, phosphorylated (p)-AKT, and MYCN proteins in prostate cancer cells, but decreased expression of these proteins was observed after DHT treatment (Fig. 6A). We also found that AKT protein expression was slightly altered after manipulation of AR signaling (Fig. 6A), supporting AKT activity being associated with activation of AR signaling [18]. In addition, C4-2 cells treated with the IFNA17 protein showed increased CHRM4 expression associated with upregulation of AKT/MYCN signaling, whereas these effects were abolished in cells with CHRM4-KD (Fig. 6B). These results suggested that IFNA17-driven AKT/MYCN signaling stimulation may be CHRM4-dependent. Although MYCN is commonly overexpressed in NEPC [19], its expression characteristics in a relatively immunosuppressive TME remain unclear. We demonstrated that MYCN-KD in NEPC-like LASCPC01 cells led to a reduction in IFNA17, which was associated with reduced mRNA levels of NE markers and PDL1, but not CTLA4 (Fig. 6C). Moreover, IFNA17-induced MYCN was associated with increased PDL1 and CTLA4 mRNA levels in C4-2 cells, whereas MYCN-KD inhibited IFNA17-driven PDL1, but not CTLA4, mRNA (Fig. 6D). These results suggested that an increase in IFNA17 was associated with abundant PDL1, and that increases in IFNA17 and PDL1 may be regulated by the MYCN transcription factor through a positive feedback mechanism. However, IFNA17-induced CTLA4 might not be regulated by the MYCN transcription factor. The MYCN transcription factor binds to a specific consensus E-box on DNA [20]. We hypothesized that ADT-induced abundance of the MYCN transcription factor in prostate cancer cells might allow it to bind to the E-box on the *IFNA17* regulatory sequence. By analyzing sequences resembling E-boxes in the putative *IFNA17* regulatory sequence region, we identified three putative E-boxes at nucleotides −4735, −4109, and −3349 relative to the *IFNA17* transcriptional start site (Fig. 6E). ChIP assays were performed in C4-2 cells in response to ADT to assess specific MYCN binding to the putative E-boxes of the *IFNA17* regulatory sequence. The consensus E-box on the *SNAI1* promoter [21] was used as a positive control. We observed increased binding activity of MYCN to E-box1, E-box2, and the positive E-box in C4-2 cells treated with the IFNA17 protein (Fig. 6F). Conversely, MYCN siRNA transfection into LASCPC01 cells showed reduced MYCN-binding activity to E-box1, E-box2, and the positive E-box (Fig. 6G). Next, reporter assays were performed using the WT E-box and a single mutated E-box (E-box1-3M) from the *IFNA17* regulatory sequence constructed into a GFP reporter plasmid (Fig. 6E). Reporter gene analysis showed that E-box1M and E-box2M, but not E-box3M, reduced CSS-driven upregulation of reporter gene activity in C4-2 cells compared to the WT E-box, while CSS-treated cells with DHT treatment showed decreased reporter activity in the WT E-box and E-box3M, but no significance was found for E-box1M or E-box2M (Fig. 6H). Importantly, IFNA17 protein-treated C4-2 cells showed increased reporter activity, but E-box1M and E-box2M exhibited reduced IFNA17 protein-driven reporter activity (Fig. 6I). We also found that E-box1M and E-box2M, but not E-box3M, abolished the ability of the ectopic MYCN cDNA vector to induce reporter activity in C4-2 cells (Fig. 6J). In LASCPC01 cells, MYCN-KD significantly reduced WT E-box reporter activity relative to NC-expressing cells, whereas E-box1M and E-box2M, but not E-box3M, abolished the MYCN-KD-driven reduction in reporter activity (Fig. 6K). These results support the hypothesis that IFNA17 expression is regulated by increased MYCN in prostate cancer cells after ADT.

### MYCN upregulates PDL1 expression associated with IFNA17 stimulation

To evaluate the IFNA17/MYCN-driven immune checkpoint pathway, we analyzed the binding capacity of the MYCN transcription factor to immune checkpoint genes (*PDL1* and *CTLA4*) by a genome browser analysis using ChIP-sequencing data (GSM2915909). Results indicated that *PDL1* has a putative MYCN-binding element at the *PDL1* transcriptional start site, but no putative MYCN-binding element was found in the *CTLA4* gene (Supplementary Fig. S2A, B). We searched for sequences resembling E-boxes in the putative *PDL1* regulatory sequence region and found three putative E-boxes located at nucleotides -6083, +15, and +452 relative to the *PDL1* transcriptional start site (Fig. 6L). C4-2 cells stimulated with IFNA17 protein showed increased MYCN transcription factor binding to E-box2 of the *PDL1* gene, but no significance was found for E-box1 or E-box3 by a ChIP assay (Fig. 6M). Conversely, LASCPC01 cells with MYCN-KD exhibited reduced MYCN binding to E-box2, but not to E-box1 or E-box3, of the *PDL1* gene (Fig. 6N). Promoter assays demonstrated that E-box2M reduced IFNA17 protein-upregulated *PDL1*/WT E-box reporter activity in C4-2 cells (Fig. 6O). MYCN-KD in LASCPC01 cells reduced MYCN-driven *PDL1*/WT E-box reporter activity, whereas E-box2M aggravated MYCN-KD-downregulated reporter activity (Fig. 6P). In summary, these findings suggested that the mechanism by which ADT-induced IFNA17 expression is associated with the abundant immune checkpoint may be regulated by the MYCN transcription factor in a positive feedback manner.

### Targeting CHRM4 suppresses tumor growth and NED of prostate cancer

**Fig. 7:**
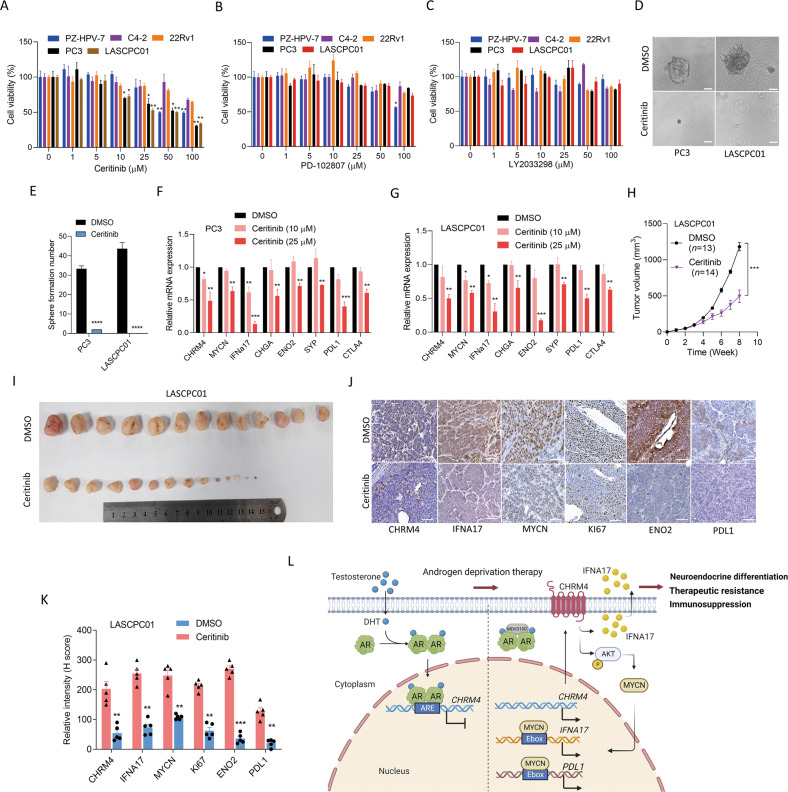
Target CHRM4 reduces the tumor growth and neuroendocrine differentiation of prostate cancer. Various prostate cancer cells were treated with 0, 1, 5, 10, 25, 50, and 100 μM of the small-molecule drugs, ceritinib (**A**), PD102807 (**B**), and LY2033298 (**C**) for 24 h, and cell viability was assessed by an MTT colorimetric assay. * vs. the control (0 μM). *n* = 8 per group. **D**, **E** Sphere-formation assay of PC3 and LASCPC01 cells treated with DMSO or 5 μM ceritinib during 1 week. * vs. DMSO. *n* = 5 per group by a *t*-test. **F**, **G** Relative CHRM4, MYCN, IFNA17, NE marker (CHGA, ENO2, and SYP), and immune checkpoint (PDL1 and CTLA4) mRNA levels in PC3 and LASCPC01 cells treated with DMSO or ceritinib at 10 and 25 μM for 24 h, as measured by an RT-qPCR analysis. * vs. DMSO, by a one-way ANOVA. Quantification of relative mRNA expressions is presented as the mean ± SEM from three biological replicates. *p < 0.05, **p < 0.01, ***p < 0.001. **H**, **I** Tumor growth monitoring of LASCPC01 cells subcutaneously injected into male nude mice. One month after injection, DMSO or ceritinib (25 mg/kg) was intraperitoneally inoculated into mice once a week for 4 weeks. The tumor volume was measured every week, and tumor tissues were collected on the last day of the experiment. DMSO-injected mice (*n* = 13); ceritinib-injected mice (*n* = 14). * vs. DMSO, ***p < 0.001, by a *t*-test. IHC staining (**J**) and representa*t*ive intensities (**K**) of CHRM4, IFNA17, MYCN, KI67, ENO2, and PDL1 in subcutaneous tumors from **I**. * vs. DMSO. **p < 0.01, ***p < 0.001. Significance was examined by a two-tailed Student’s *t*-test. **L** A schematic summary of this study. Our study focused on androgen-deprivation therapy (ADT)-induced NE prostate cancer (NEPC) to determine the mechanism by which androgen receptor (AR) loss of function might promote CHRM4-driven AKT/MYCN signaling leading to increased IFNA17 and PDL1 expressions. Increased abundances of IFNA17 and PDL1 may be regulated by the MYCN transcription factor through a positive feedback mechanism. Serum IFNA17 levels can be considered a prognostic biomarker in NEPC-like prostate cancer, and targeting CHRM4 may have the potential to inhibit NEPC progression.

Targeting muscarinic receptors for illness therapy was established in several studies; for instance, CHRM3 antagonists (darifenacin and tiotropium) decreased lung and colon cancer proliferation [22]. Pharmacological inhibition of CHRM4 by the antagonist, PD102807, was found to promote the burst-forming unit erythroid, a critical cell type for treating anemias [23]. We conducted a molecular docking analysis and in-house drug screening to study the promising therapeutic inhibitory effects of approved drugs targeting CHRM4 in prostate cancer. After the screening, we selected a CHRM4 candidate inhibitor, ceritinib, to test its pharmacological effects on prostate cancer cells compared to known CHRM4 inhibitors (LY2033298 and PD102807) (Table 1). Interestingly, AR-negative PC3 and NEPC-like LSCPC01 cell lines were significantly sensitive to ceritinib compared with normal prostate epithelial cells or AR-positive cancer cells, whereas LY2033298 and PD102807 had no effect on prostate cancer cells (Fig. 7A–C). To validate the relative rate of cell viability, we evaluated the functional relevance of the tumorsphere-formation efficiency in PC3 and LASCPC01 cells and found that ceritinib inhibited sphere formation in both cell lines relative to DMSO-treated cells (Fig. 7D, E). Furthermore, PC3 and LASCPC01 cells treated with ceritinib exhibited reductions in CHRM4, MYCN, IFNA17, NE markers, and immune checkpoints (Fig. 7F, G), suggesting that targeting CHRM4 may suppress NED and immune checkpoint pathways. In order to test the efficacy of ceritinib against NEPC-like tumor growth in vivo, mice were subcutaneously injected with LASCPC01 cells and treated with ceritinib after tumor formation. We found that mice harboring LASCPC01 tumor cells treated with ceritinib showed a significant decrease in tumor growth compared to control mice (Fig. 7H, I). Interestingly, we found that the reduction of CHRM4 protein in mice treated with ceritinib was associated with the reduction of IFNA17, MYCN, KI67, ENO2 and PDL1 proteins by IHC staining, compared to mice treated with DMSO (Fig. 7J, K). These results suggest that CHRM4-targeting therapy might suppress a variety of growth rates as well as NED properties of NEPC-like prostate cancer cells. In summary, our results demonstrated that a regulatory mechanism that inhibits AR signaling in primary prostate cancer through ADT might inhibit the tumor-suppressive role of AR, leading to the activation of CHRM4/AKT/MYCN signaling to promote IFNA17 secretion in the TME, which may be involved in stimulating immune checkpoint pathway components in a subset of prostate cancer patients with NE characteristics (Fig. 7L).

**Table 1:** Computed binding affinity of CHRM4 with approved drugs.

Rank	Name	Indication	Estimated binding energy (lower is better)
1	Ceritinib	For treatment of anaplastic lymphoma kinase (ALK)-positive metastatic non-small-cell lung cancer (NSCLC)	−153.867
2	Gentamicin	For treatment of serious infections caused by susceptible strains	−144.724
3	Manidipine	For the treatment of hypertension	−143.059
4	Hygromycin B	For bacteria, fungi and higher eukaryotic cells infection	−143.033
5	Indinavir	For treatment of HIV infection	−141.669
6	Crocin	treatment of Hyperglycemia	−140.394
7	Kanamycin	For treatment of infections	−139.528
8	Osimertinib	For treatment of patients with metastatic epidermal growth factor receptor (EGFR) T790M mutation-positive non-small-cell lung cancer	−138.996
9	Ambrisentan	For treatment of idiopathic (primary) pulmonary arterial hypertension (IPAH) and pulmonary arterial hypertension (PAH)	−137.94
10	Deflazacort	For treatment of Duchenne Muscular Dystrophy	−134.921
11	Methotrexate	For treatment of pediatric acute lymphoblastic leukemia	−134.681
12	Ambenonium	For treatment of muscle weakness due to muscle disease	−134.659
13	Hexoprenaline	For treatment of bronchoconstriction	−134.494
14	Pemetrexed	For treatment of malignant pleural mesothelioma	−134.433
15	Pipazetate	For the treatment of cough	−134.426
16	Cromoglicic acid	For treatment of patients with bronchial asthma	−134.413
17	Pralmorelin	For growth hormone deficiency	−134.123
18	Ombitasvir	For treatment of patients with genotype 4 chronic hepatitis C virus	−132.991
19	Pranlukast	For treatment of Allergic rhinitis or Asthma	−132.973
20	Trospium	For the treatment of overactive bladder	−132.796
33	Tiotropium	mAchR inhibitor	−130.224
2744	LY2033298	mAchR4 modulator	−89.8963
3353	PD102807	mAchR4 inhibitor	−84.3893
4473	Acetylcholine	Natural mAchR substrate	−62.3897

5DSG was chosen as docking module which binding pocket of embedded tiotropium was set as binding site. Molecular docking was performed by iGemDock v2.1 which population, generations, and number of solution were 200, 70, 3, respectively.

## Discussion

The primary goal of ADT is to suppress AR transcriptional activity, but many patients are unable to maintain stable inhibition with prolonged treatment, and AR transcriptional activity eventually changes regardless of androgen castration levels [24]. Although AR transcriptional activity can be restored in CRPC [25], a mouse study found that long-term androgen deprivation in prostate cancer tumors responds to androgens and leads to slower tumor progression [26]. Moreover, therapy with high-dose testosterone was evaluated in a small cohort of 12 patients with prolonged castration, and the majority of patients adapted well, resulting in the repression of tumor growth and safety for patients [27]. The retinoblastoma (Rb) protein combines with E2F transcription factors to form a repressor complex [28]. Previous research demonstrated that the AR could recruit hypophosphorylated Rb to DNA replication gene loci and enhance the suppressive function of the Rb-E2F complex [16]. The tumor-suppressive efficacy of high-dose testosterone was mediated by the important role of the Rb/p130-E2F complex and strengthened by CDK4/6 inhibitor treatment [16]. Androgens are capable of promoting and suppressing prostate cancer progression [29]. In this study, we demonstrated an association between AR inhibition and increased CHRM4 expression in various prostate cancer cell lines and clinical datasets. Our findings suggest that androgen-activated AR can downregulate CHRM4 expression by directly binding to and suppressing *CHRM4*. Overexpression of CHRM4 in prostate cancer cells after ADT may promote migration, invasion, and proliferation, especially in the progression of NED. AR expression in normal prostate tissues may inhibit CHRM4 expression; however, AR inhibition in advanced prostate cancer after ADT may lead to CHRM4/AKT/MYCN activation, which in turn promotes the NED and invasiveness of prostate cancer cells. Our findings support the hypothesis that the AR is a tumor suppressor in prostate cancer.

Recent investigations revealed that immune cells in the TME play crucial roles in mediating NEPC formation [30]. Tumor cells can induce long-term immune responses and interact with immune cells to promote metastasis and NEPC development [30]. Owing to limitations of conventional ADT, immunotherapies have been anticipated as optimal alternatives for prostate cancer patients [31]. However, enormous challenges remain for scientists and patients because of the immunosuppressive elements in the prostate cancer TME and difficulties posed by interactions between microenvironmental variables [30]. We found a correlation between CHRM4 and IFNA17 cytokine response signaling in the TME of prostate cancer, in which abundant IFNA17 was found in prostate cancer cells cocultured with CM collected from M2-type TAMs.

Tumor-infiltrating lymphocytes, which can be M2-type TAMs, produce anti-inflammatory cytokines and have the capacity to suppress the immune system and promote prostate cancer formation [31]. Bone morphogenetic protein (BMP)-6 released by prostate cancer cells stimulates M2-type-TAMs to produce IL-6 [32], while tumor-infiltrating M2-type-TAMs promote NED development by activating downstream IL-6 signaling [33]. We demonstrated that CHRM4/IFNA17-activated prostate cancer cells might interact with M2-type TAMs to promote NED and immune checkpoint pathways in prostate cancer cells, contributing to the development of an immunosuppressive TME and NEPC. CTLA4 is an immune checkpoint expressed by regulatory T (Treg) cells that can bind to cluster of differentiation 80 (CD80) and CD86 on dendritic cells with high affinity [34]. Moreover, CTLA4 modulates the function of antigen-presenting cells to reduce T-cell functions [35]. PDL1 expression was found in a small subset of primary prostate cancer cases, but was higher in metastatic CRPC in a clinical evaluation at Johns Hopkins Hospital [36]. Our findings show that PDL1, but not CTLA4, may be positively regulated by the MYCN transcription factor driven by the CHRM4/IFNA17 axis. MYCN-driven PDL1 was significantly elevated when prostate cancer cells were cultured under ADT conditions or with IFNA17 treatment, and its expression decreased after CHRM4-KD. Our findings suggest that ADT-induced CHRM4 may activate AKT/MYCN signaling to induce IFNA17 secretion and upregulate immune checkpoints in the TME to drive potential immunosuppressive responses, leading to NED and metastasis in prostate cancer.

IFN-α is a member of the type I INF family that is released by host cells and immune cells, and is capable of fighting pathogens, viruses, bacteria, and tumor cells by causing nearby cells to promote protective defenses [37]. The anti-hepatitis C virus (HCV) activity of IFN-α subtypes was evaluated by treating infected liver cancer cells with IFN-α. Three subtypes, IFNA17, IFNA7, and IFNA8, showed stronger action against HCV than IFNA2a [38]. The IFNA17 184Ile allele is associated with an increased risk of cervical cancer, suggesting that IFNA17 polymorphisms may be key biomarkers of cervical cancer susceptibility [39]. In our study, the advanced CRPC group had significantly higher IFNA17 levels than did the primary prostate cancer group, and both cancer groups had higher IFNA17 levels than the BPH group. This was consistent with the abundance of CHRM4 and NE markers in CRPC samples. We demonstrated that IFNA17 overexpression might enhance functional characteristics, such as migration, proliferation, and association with elevated levels of CHRM4, NE markers, and immune checkpoints in prostate cancer cells. Our study evaluated serum IFNA17 levels in patients with prostate cancer. It may be worthwhile to further investigate IFNA17 as a potential biomarker with human serum diagnostic tools and the relationship between CHRM4 levels and clinical stages of advanced prostate cancer.

## Conclusions

In summary, we found that ADT-induced IFNA17 expression positively affected the elevation of NED and immune checkpoint abundances in prostate cancer cells, which was dependent on CHRM4. We established a link between elevated IFNA17 levels and NED-related immunosuppressive responses through CHRM4/AKT/MYCN activation after resistance to ADT therapy. Inhibition of AR signaling by androgen withdrawal or AR antagonists may disrupt AR function, leading to an increased CHRM4/AKT/MYCN axis and immune checkpoint pathway activation. Analysis of CHRM4-driven IFNA17 cytokine release in the prostate cancer TME following ADT resistance may provide a clear understanding of the feedback loop consisting of CHRM4/AKT/MYCN in the context of AR inhibition.

## Materials and methods

### Cell culture and reagents

AR-positive prostate cancer cell lines (LNCaP, C4-2, and 22Rv1) and an AR-negative prostate cancer cell line (PC3) were obtained from ATCC and cultured in RPMI-1640 medium (Thermo Fisher Scientific, 11875-085) supplemented with 5% fetal bovine serum (FBS; EMD Millipore, TMS-013-BKR) and 1% penicillin. NEPC-like LASCPC01 cells were obtained from ATCC and cultured in RPMI-1640 medium supplemented with 10 nM hydrocortisone (Sigma-Aldrich, H0888), insulin/transferrin/selenite (Thermo Fisher Scientific, 41400-045), 200 nM β-estradiol (Sigma-Aldrich, E2758), 5% FBS, and 1% penicillin. The PZ-HPV-7 normal prostate epithelial cell line was purchased from ATCC and cultured in keratinocyte serum-free medium (K-SFM; ThermoFisher, 17005-042) supplemented with 0.05 mg/mL bovine pituitary extract (BPE; ThermoFisher) and 5 ng/mL human recombinant epidermal growth factor (EGF; ThermoFisher). C4-2-MDVR cells are an ADT-resistant cell line generated from C4-2 cells maintained for 6 months in RPMI-1640 medium with 5% FBS and 20 μM MDV3100. All cell lines were routinely checked for mycoplasma contamination using a Mycoplasma PCR Detection Kit (Omicsbio, G238) within 6 months before the experiments. To mimic ADT, cells were cultured in 10% charcoal-stripped serum (CSS; ThermoFisher, 12676-029)-containing RPMI-1640 medium under standard culture conditions. Treatment with an AR antagonist was performed using 20 μM enzalutamide (MDV3100; Selleckchem, S1250). Treatment with the AR ligand was administered using 10 nM dihydrotestosterone (DHT; Selleckchem, S4757) for 24 h. Candidate CHRM4 inhibitors (ceritinib, LY2033298, and PD102807) were purchased from MedChemExpress, and concentrations of each candidate medicine for the cell viability assay were 0, 1, 5, 10, 25, 50, and 100 μM for 24 h.

### Proliferation assay

C4-2 cells stably transfected with an empty vector (EV), CHRM4, or IFNA17 cDNA vector, and PC3 cells stably transfected with a non-targeting control (NC) or CHRM4 shRNA vector were seeded in 96-well plates at a density of 3 × 10^3^ cells/well. The proliferation rate was assessed every 24 h for 5 days. Cells were stained daily with 0.5% crystal violet for 15 min, washed four times with distilled water, and then dried. Before measurement, crystal violet was completely dissolved by adding 100 μL of 50% ethanol containing 0.1 M sodium citrate to each well of the plate with gentle shaking. Absorbances at two wavelengths of 540 and 405 nm were determined using a microplate reader. The experiment was performed in multiple wells at each time point, and average values were recorded.

### Migration and invasion assays

For the migration assay, 3 × 10^4^ cells/well were suspended in serum-free medium and added to 24-well Boyden chambers (8-μm pore size), and the chambers were placed in a 24-well culture plate. For the invasion assay, Boyden chambers were precoated with 200 μg/mL Matrigel matrix (Corning, 354234). Matrigel-coated transwell chambers were prepared by adding 200 μL of serum-free medium diluted with Matrigel. The lower chambers were filled with 600 μL of complete medium, and then whole plates were incubated for 24 h for migration and 12 h for invasion at 37 °C with 5% CO_2_ and standard cell culture conditions. The transwell chambers were then fixed with methanol for 5 min before being dyed with 0.5% crystal violet for 15 min. After washing with distilled water, non-invading cells in the chambers were removed with a cotton swab, while invading cells remained on the underside of the membranes. The chambers were dried at room temperature, and a phase-contrast microscope (Olympus) was used to acquire images of migratory or invasive cells on the underside of the membranes, with three replicates being counted each time.

### Sphere-formation assay

For the sphere-formation assay, 500 cells/well were prepared in complete medium and then combined with the required amount of standard Matrigel matrix (Corning, 354234). The mixture was added to the bottom edge of a six-well plate and incubated overnight for aggregation. The following day (day 0), 2 mL of the culture medium was added to each well and cultured for 7 days. To assess the cytotoxicity of the candidate CHRM4 inhibitor, PC3 and LASCPC01 cells were treated with 5 μM ceritinib for 7 days. Tumorspheres were observed in each well, and photos were taken with a phase-contrast microscope (Olympus) and counted.

### Tumorigenicity assays in mice

The protocols of this assay were based on *Guidelines for Care and Use of Laboratory Animals* by the Council of Agriculture, Executive Yuan, Taiwan, and approved by the Taipei Medical University Institutional Animal Care and Use Committee (approval ID: LAC-2021-0526). Under double-blind conditions, four 6-week-old male nude mice (Academia Sinica, Taipei, Taiwan) were subcutaneously injected with 10^6^ PC3/NC or PC3/shCHRM4 cells into the right flank of each group. Cells were suspended in 100 μL of a mixture of 50% Matrigel matrix and 50% complete medium. For ceritinib treatment, 2 weeks after the subcutaneous injection of 10^6^ cells/site of LASCPC01 cells, mice received an intraperitoneal injection of 2.5 mg/kg ceritinib or DMSO (control) once weekly. Tumor sizes and mouse body weights were measured weekly for 8 weeks. Mice were sacrificed via CO_2_ anesthetization, and tumors were collected, weighed, sliced, and IHC-stained for CHRM4, IFNA17, MYCN, KI67, ENO2, CHGA, and PDL1. All of the antibodies used for IHC staining are listed in Supplementary Table S4. The tumor volume (V) was calculated using the following formula: *V* = 0.5236 × *H* × *W* × *L*, where *H*, *W*, and *L* are the height, width, and length, respectively.

### Enzyme-linked immunosorbent assay (ELISA)

Sera from patients with BPH (23 samples), primary prostate cancer (16 samples), and CRPC (eight samples) were collected from Taipei Medical University-Wan Fang Hospital (Taipei, Taiwan). Written informed consent was obtained from all patients, and the study was approved by the Taipei Medical University Joint Institutional Review Board (approval no. N202201101), in accordance with the *Declaration of Helsinki*. After drawing blood, whole-blood tubes were allowed to sit for 30 min to clot, and then serum was centrifuged for 20 min at 1000 × *g*. To eliminate particles from cell culture supernatants, the supernatants were centrifuged for 20 min at 1000 × *g*. Serum samples were divided into aliquots and stored at −80 °C until further use. Samples were then thawed twice. IFNA17 levels were measured using a human IFNA17 ELISA kit (Biobool/E020241 for cell lines and MyBiosource/MBS9311549 for human serum). According to the manufacturer’s instructions, the average of duplicate readings for each standard, control, and sample was subtracted from the average zero-standard optical density. Using computer software capable of constructing a four-parameter logistic fit curve, a standard curve was generated, and the level of the samples was then calculated.

### Immunohistochemical (IHC) staining

Prostate tumor samples selected from patients with BPH, primary prostate cancer, and CRPC were collected from the Taipei Medical University Biobank. The study protocol was approved by the Taipei Medical University Joint Institutional Review Board (approval no. N202201101). Before IHC staining, tumor slices were deparaffinized, rehydrated, and heated. Slices were then stained with primary antibodies, as described in Supplementary Table S4, including CHRM4 and CHGA. Tris-buffered saline (TBS) buffer with 0.1% Triton X-100, conjugated with avidin, and colored with 3.3′-diaminobenzidine reagent was used as the washing solution. After washing, slices were stained with a secondary antibody, dried, and mounted with glycerol. Pathological diagnoses and intensities were determined by a pathologist (Wei-Yu Chen). For the histomorphometric analysis of tissue sections, 10 bright-field microscopic images of IHC-stained sections were captured in each core using a phase-contrast microscope at 200× magnification (Olympus IX73). The intensity of the targets was defined as 0 (negative), 1+ (weakly positive), 2+ (moderately positive), and 3+ (strongly positive). The range of intensity scoring values varied from 0 to 300 and were determined by the following formula:1 × (% of 1+ cells) + 2 × (% of 2+ cells) + 3 × (% of 3+ cells).

### Chromatin immunoprecipitation (ChIP) assay

A ChIP assay was performed using an EZ-Magna ChIP^TM^ IP kit A (Sigma-Aldrich, 17-10086) following the protocol in the manufacturer’s instructions. After treatment, 10^6^ cells were fixed with 1% paraformaldehyde/complete medium for 10 min followed by termination of fixing by incubation with 125 mM glycine buffer for 5 min. Fixed cells were washed with phosphate-buffered saline (PBS) containing proteinase and phosphatase inhibitors in a freezer. Cells were then scraped off into PBS buffer, and the debris was collected. Chromatin within the debris was released using lysis buffer in the kit and disrupted into 150-bp pieces by sonication (Qsonica). Chromatin-protein complexes were labeled with 10 ng of an anti-AR antibody (Sigma-Aldrich, 06-680), anti-MYCN antibody (Abcam, ab16898), anti-acetyl-histone H3 antibody (positive control, Novus, NB300-221), or normal rabbit immunoglobulin G (IgG) (negative control, Santa Cruz, sc-2027), followed by enrichment using protein A-coated magnetic beads. Chromatin was released from the complexes by proteinase K (Sigma-Aldrich, 124568) following heat inactivation, and was identified by a reverse-transcription quantitative polymerase chain reaction (RT-qPCR). The ChIP antibodies and qPCR primers used are listed in Supplementary Table S5. For the ChIP-sequencing analysis, ChIP-sequencing data were downloaded from the Gene Expression Omnibus (GEO) (GSE84432) and analyzed by the Genome Brower (Genomics Institute, University of California at Santa Cruz, CA, USA).

### Promoter reporter assay

AR response elements (AREs) are located upstream of human *CHRM4* on chromosome 11:46397008 (ARE1: +959) and 46394989 (ARE2: −1950) at GRCh38. E-boxes of human *IFNA17* are located on chromosome 9:21232509 (E-box1: −4735), 21233136 (E-box2: −4109), and 21233899 (E-box3: −3349) at GRCh38. E-boxes of human *PDL1* are located on chromosome 9:5444421 (E-box1: −6083), 5450521 (E-box2: +15), and 5450959 (E-box3: +452) at GRCh38. These regulatory sequences with response-element green fluorescence protein (GFP) reporter vectors (pGreenFire1-ISRE Lentivector; System Biosciences, TR016PA-P) were constructed using the Clone-it Enzyme free Lentivector Kit (System Biosciences). Cells (5 × 10^4^ cells/well) in 12-well plates were transiently transfected with 1 µg of the wild-type (WT) and mutant (M)-*CHRM4*-GFP reporters containing AREs using the X-tremeGENE™ HP DNA transfection reagent. To mimic ADT, reporter plasmid-transfected cells were treated with CSS-containing medium or 20 μM MDV3100 for 48 h. AR-ligand-treated cells were treated with 10 nM DHT for 48 h. The WT- and M-GFP reporters were co-transfected with an EV, AR, or MYCN-expressing vector in cells or co-transfected with the NC, AR, or MYCN siRNA in cells. Promoter function was analyzed using fluorescence-activated cell sorting (FACS, BD Biosciences), and relative median fluorescent intensity (MFI) values were measured for GFP by FACS using FACSDiva software (BD Biosciences) and normalized to the value of the vehicle. Three independent experiments were performed in triplicate.

### THP-1 differentiation

Human monocyte THP-1 cells were obtained from ATCC, counted, and seeded in 10-cm plates at a concentration of 3 × 10^6^ cells/ml in 10 mL RPMI medium containing 10% FBS supplemented with 0.05 mM 2-mercaptoethanol (Merck, M6250) and maintained at 37 °C in a 5% CO_2_ incubator prior to stimulation. All macrophage-polarized conditions used 5 μg/ml phorbol 12-myristate 13-acetate (PMA; Merck, P8139) added to RPMI media for 24 h to prime THP-1 monocytes into macrophage-like cells, followed by washing the PMA off and a 72-h rest period in fresh medium prior to exposure to cytokines. M1 was polarized with 100 ng/ml lipopolysaccharide (LPS) (Invitrogen, O111:B4) and 30 ng/ml IFN-γ (Croyez, C01080-GMP-100), M2a was polarized with 30 ng/ml IL-4 (Sino, 11846-HNAE), and M2c was polarized with 30 ng/ml IL-10 (MCE, HY-P7030A). The cytokine exposure time was maintained at 48 h. THP-1-polarized conditioned media were collected and centrifuged for 5 min at 1000 × *g* to remove particulates. For the coculture experiment, C4-2 cells were incubated with different concentrations (1:10, 1:20, or 1:50) of THP-1 polarization-conditioned medium for 48 h.

### Statistical analysis

All experiments were performed at least thrice. GraphPad Prism software vers. 8.0 was used to construct each plot, and results are presented as the mean and standard error of the mean (SEM). A one-way analysis of variance (ANOVA), two-way ANOVA, two-tailed *t*-test, and Bonferroni’s post-hoc test were used to establish statistical significance between compared groups. IHC staining of tissue samples was compared using paired two-tailed Student’s *t*-tests. Cutoff values were predetermined by half the number of patients in the z-score analyses, and *p* values of <0.05 were regarded as statistically significant.

### Supplementary information

Supplemental File 1

Supplemental File 2

Reproducibility checklist

## Data Availability

Data used in the current study are available from the corresponding author upon reasonable request.
