# Correction: CHRM4/AKT/MYCN upregulates interferon alpha-17 in the tumor microenvironment to promote neuroendocrine differentiation of prostate cancer

**DOI:** 10.1038/s41419-023-06363-1

**Published:** 2024-01-29

**Authors:** Yu-Ching Wen, Van Thi Ngoc Tram, Wei-Hao Chen, Chien-Hsiu Li, Hsiu-Lien Yeh, Phan Vu Thuy Dung, Kuo-Ching Jiang, Han-Ru Li, Jiaoti Huang, Michael Hsiao, Wei-Yu Chen, Yen-Nien Liu

**Affiliations:** 1 Department of Urology, Wan Fang Hospital, Taipei Medical University, Taipei, 11696 Taiwan; 2 Department of Urology, School of Medicine, College of Medicine, Taipei Medical University, Taipei, 11031 Taiwan; 3 TMU Research Center of Urology and Kidney, Taipei Medical University, Taipei, 11031 Taiwan; 4 International PhD Program in Medicine, College of Medicine, Taipei Medical University, Taipei, 11031 Taiwan; 5 Graduate Institute of Cancer Biology and Drug Discovery, College of Medical Science and Technology, Taipei Medical University, Taipei, 11031 Taiwan; 6 Genomics Research Center, Academia Sinica, Taipei, 11529 Taiwan; 7 Department of Pathology, Duke University Medical Center, Durham, NC 27710 USA; 8 Department of Pathology, Wan Fang Hospital, Taipei Medical University, Taipei, 11696 Taiwan; 9 Department of Pathology, School of Medicine, College of Medicine, Taipei Medical University, Taipei, 11031 Taiwan; 10 TMU Research Ceśnter of Cancer Translational Medicine, Taipei Medical University, Taipei, 11031 Taiwan

**Keywords:** Cancer microenvironment, Genetics research, Prognostic markers, Prostatic diseases

Correction to: *Cell Death and Disease* 10.1038/s41419-023-05836-7, published online 04 May 2023

In this article, the author Yen-Nien Liu has been affiliated with:

^4^International PhD Program in Medicine, College of Medicine, Taipei Medical University, Taipei 11031, Taiwan

^10^TMU Research Center of Cancer Translational Medicine, Taipei Medical University, Taipei 11031, Taiwan.

it should read:

^5^Graduate Institute of Cancer Biology and Drug Discovery, College of Medical Science and Technology, Taipei Medical University, Taipei 11031, Taiwan.

^10^TMU Research Center of Cancer Translational Medicine, Taipei Medical University, Taipei 11031, Taiwan.

The original article has been corrected.

