# Supplementary material for: CHRM4/AKT/MYCN upregulates interferon alpha-17 in the tumor microenvironment to promote neuroendocrine differentiation of prostate cancer
Source: Cell Death Dis. 2023 May 4;14(5):304. doi: 10.1038/s41419-023-05836-7 (PMC10160040; doi:10.1038/s41419-023-05836-7)
Supplement: Supplementary file 1 — Supplemental File 1 [file 41419_2023_5836_MOESM1_ESM.docx]

**Supplementary File**

**Supplementary** **Materials and Methods**

**Reagents, and constructs**

Overexpression of the AR, CHRM4, IFNA17, and MYCN was generated by establishing a pCDH-CMV-MCS-EF1-Puro vector (System Biosciences, Palo Alto, CA, USA) encoding AR, CHRM4, IFNA17, and MYCN full-length complementary (c)DNA; an empty vector (EV) was used as a control. Small interfering (si)RNAs (NC, siAR, and siMYCN) were obtained from ON-TARGETplus SMARTpool siRNA (D-001810, L-003400, and L-003913; Thermo Scientific Dharmacon, Waltham, MA, USA). Knockdown (KD) of CHRM4 or IFNA17 was generated by infection with a recombinant lentivirus encoding a human CHRM4 or IFNA17 short hairpin (sh)RNA vector (RNAi Core Lab, Taipei, Taiwan); a non-target control (NC) pLKO_TRC005-Puro vector was used as a control. Regulatory sequence reporters of the *CHRM4,* *IFNA17,* and *PDL1* genes were constructed using the pGreenFire reporter (System Biosciences), and a Site-Directed Mutagenesis System kit (Invitrogen, Waltham, MA, USA) was used for response element mutations. All primers used to generate these constructs are listed in Supplementary Table S1. All constructs were verified by a DNA sequence analysis.

**Reverse-transcription (RT)-quantitative polymerase chain reaction (qPCR)**

An RNeasy Midi Kit (Qiagen, 74004) was used to isolate total messenger (m)RNA. For RT, 1 μg of total mRNA was used with the iScript^TM^ cDNA Synthesis Kit (Bio-Rad, 1708890). The iTaq Universal SYBR Green Supermix (Bio-Rad, 1725120) was employed for amplification. All primer pairs were reacted using a thermocycler with initial incubation at 95 °C for 10 min, followed by 40 cycles at 95 °C for 15 s and 60 °C for 1 min. All reactions were standardized to the expression of human 18S ribosomal (r)RNA and performed in triplicate. All primers of the qPCR are described in Supplementary Table S2.

**Western blot analysis**

Cells were lysed in 200 μL RIPA buffer (ThermoFisher Scientific, 8900) with a protease inhibitor (Roche, 11697498001) and phosphatase inhibitor cocktail (Roche, 4906845001). Protein samples were measured using the Bradford reagent (Bio-Rad, 5000006) and separated by sodium dodecylsulfate polyacrylamide gel electrophoresis (SDS-PAGE). After gels were transferred to polyvinylidene difluoride (PVDF) or nitrocellulose membranes (ThermoFisher Scientific), the membranes were blocked with 5% bovine serum albumin in Tris-base buffer containing 0.1% Tween-20 for 1 h. Then, primary antibodies were added and incubated at 4 °C overnight, and samples were placed on a gentle shaker. After washing three to five times, secondary antibodies were added and incubated at room temperature for 1 h. All of the information of antibodies of the Western blot analysis are described in Supplementary Table S3. Protein bands were detected using enhanced chemiluminescence (ECL) plus Western Blot Detection Reagents (Millipore, WBULS0100).

**3-(4,5-Dimethylthiazol-2-yl)-2,5-diphenyltetrazolium bromide (MTT) assay**

Cell viability for CHRM4 candidate inhibitor treatment was analyzed with an MTT assay (Santa Cruz Biotechnology), according to the manufacturer’s protocol. For the experiment, a single-cell suspension of 500 cells/well in multiple wells was assessed at each time point and averaged. Absorbance was quantified at a wavelength of 570 nm using a CLARIOstar microplate reader (BioTek). Relative cell numbers are presented as the ratio of OD 550 nm normalized to day 0.

**Dataset analysis**

To compare IFNA17 expression levels with prostate cancer progression and survival, we used mRNA expression data from GSE21032 ^1^, The Cancer Genome Atlas (TCGA) ^2^, and Beltran ^3^ human prostate cancer datasets. Expression data were log2-normalized. Gene set enrichment analysis (GSEA) software and gene signatures of androgen-upregulated (GO, PID, Hallmark, Wang ^4^, and Nelson ^5^) and immune-responsive (GO, KEGG, and Biocarta) datasets were downloaded from the Broad Institute ^6^ and used to determine correlations with CHRM4 and IFNA17 levels. A normalized enrichment score (NES) and false discovery rate (FDR) were calculated using the GSEA program. Cutoff values used to identify “CHRM4 high” or “IFNA17 high” patients were predetermined by half the number of patients from the GSEAs. Tumors were mean-stratified by IFNA17 expressions, and the mean expression of each gene was determined in each group. For overall survival, the study used the GSE21032 dataset ^1^, which was accessed from the Memorial-Sloan Kettering Cancer Center (MSKCC) Cancer Genomics data portal (http://cbio.mskcc.org/cancergenomics/prostate/data/), from which we downloaded clinical and publicly available gene expression data of 98 primary and 13 metastatic prostate cancer samples. In total, 110 patients (98 primary and 13 metastatic prostate cancer samples) were divided into two groups according to their mean IFNA17 expression: high IFNA17 expression (IFNA17 high, *n*=56) and low IFNA17 expression (IFNA17 low, *n*=55).

**In-house drug screening**

Drug screening was performed through molecular docking using iGemDock V2.1 ^7^. According to the protein information from Uniprot, CHRM4 was built using homology modeling by SWISS-MODEL ^8^. As protein modules were collected, water and internal ligands/inhibitors were deleted, and hydrogen was re-filled. The docking center of CHRM4 was set to Asn^423^ which is the core of the selective binding site to acetylcholine ^9^. A molecular model of approved drugs was obtained from the ZINC database and redundant compounds, endogenous metabolites, compounds not for sale, drugs for external uses, anodynes, and magnetic resonance imaging contrast agents were excluded (a total of 6384 compounds) ^10^. The highest docking simulation of each compound was selected as a putative CHRM4 inhibitor, and ranked according to the calculated free energy.

**Supplementary Figures**

**
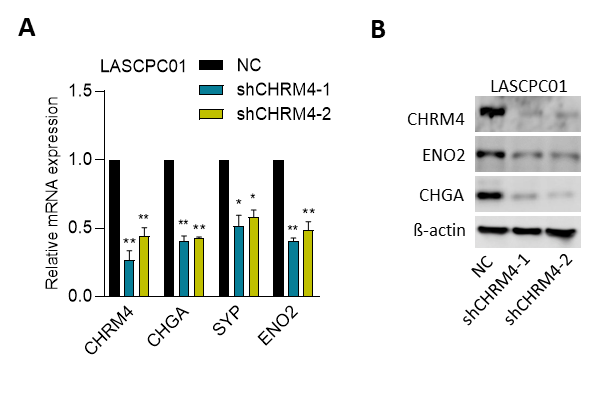
**

**Supplementary Fig. S1:** **Knockdown of CHRM4 reduces neuroendocrine differentiation of LASCPC01 cells. A** CHRM4 and NE marker (CHGA, SYP, and ENO2) mRNA levels in LASCPC01 cells stably transfected with a non-target control (NC) or CHRM4 shRNA vector, measured by an RT-qPCR analysis, * vs. the NC (**A**), by a one-way ANOVA. Quantification of relative mRNA levels are presented as the mean ± SEM from three biological replicates. * *p*<0.05, ** *p*<0.01, *** *p*<0.001. **B** CHRM4 and NE marker protein levels in LASCPC01 cells expressing NC or CHRM4 shRNA vector.

**
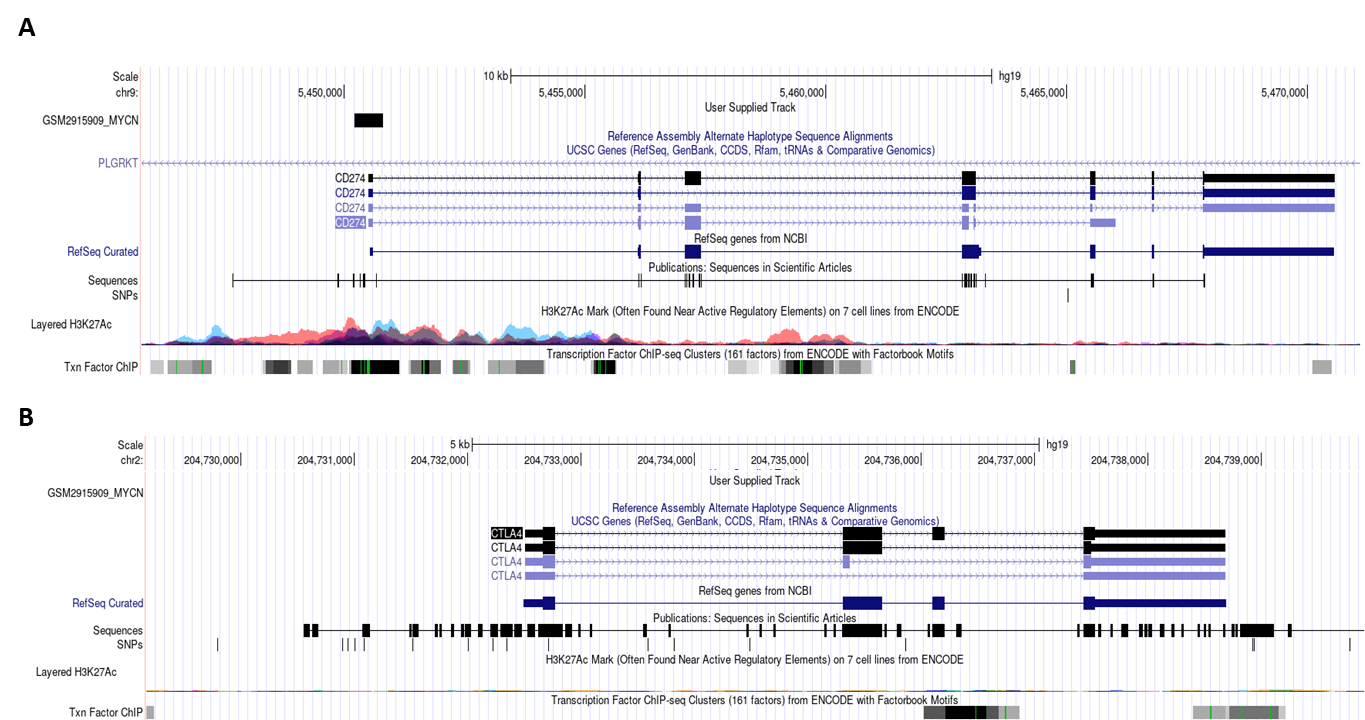
**

**Supplementary Fig. S2:** **MYCN may directly bind to the promoter of *PDL1* but not to *CTLA4*.** **A, B** ChIP-sequencing analysis of the detected DNA-binding sites for MYCN of the *PDL1* (**A**) and *CTLA4* (**B**) genes in Kelly cells as labeled as black boxes in the tracks. ChIP-sequencing data were downloaded from the Gene Expression Omnibus (GEO) (GSM2915909) and analyzed by Genome Brower (Genomics Institute, UCSC, CA, USA).

**Supplementary Tables**

Primer sequences of the wild-type (WT) androgen receptor (AR)- or MYCN-binding elements of human *CHRM4, IFNA17,* or *PDL1* regulatory sequence reporters and the AR- or MYCN-binding element mutants of the human *CHRM4, IFNA17,* or *PDL1* regulatory sequence reporters are listed 5’-3’. M, mutant; F, forward; R, reverse.

| Promoter reporter | Sequence |
| --- | --- |
| *CHRM4*/AREWT F | TGGCTGACTGATTACT |
| *CHRM4*/AREWT R | ATGCCAGCCGGCGTGGCA |
| *CHRM4*/ARE1M F | AGGCAGTGCCACCCAGCCTTCCAAGGAACGC |
| *CHRM4*/ARE1M R | GGGTGGCACTGCCCTGAGCTGGAC |
| *CHRM4*/ARE2M F | GAACCAAGGATTTAGCCTTGGAGAACTTGAA |
| *CHRM4*/ARE2M R | AAATCCTTGGTTCGTAGGAGGA |
| *IFNA17*/EBOXWT F | TGAAACAGTGAAGATGA |
| *IFNA17*/EBOXWT R | AAGGAGAGATTCTTCCCATT |
| *IFNA17*/EBOX1M F | ACTTTTTGGTAGAGCATTCCTGCTGTGCTGG |
| *IFNA17*/EBOX1M R | CTCTACCAAAAAGTGGCCAGTC |
| *IFNA17*/EBOX2M F | CCATGCATGCCTGAGCATTCCCTCTGCTGAAA |
| *IFNA17*/EBOX2M R | CTCAGGCATGCATGGGTA |
| *IFNA17*/EBOX3M F | ACAGAGTGCTCCTCTTTCCCCTGGAACATGA |
| *IFNA17*/EBOX3M R | AGGAGCACTCTGTGGGGTAGA |
| *PDL1*/EBOXWT F | ATAAATAAATATACATTA |
| *PDL1*/EBOXWT R | AGATATATATGCAATGAGTT |
| *PDL1*/EBOX1M F | GGTGGGCTTCTGGATGGGGTTTGGTCACAAGAATGACCAA |
| *PDL1*/EBOX1M R | CCATCCAGAAGCCCACCAAGTTACTTCA |
| *PDL1*/EBOX2M F | CCTGGCGCAACGCTGAGCGTTTGGCGCGTCCCGC |
| *PDL1*/EBOX2M R | CTCAGCGTTGCGCCAGGCCCGGA |
| *PDL1*/EBOX3M F | GGTCCAAGTCCACCGCCGTTTGCTTGCTAGTAACA |
| *PDL1*/EBOX3M R | GCGGTGGACTTGGACCCGTGTCCA |

**Supplementary Table S2. RT-qPCR primer sequences.** Sequences of the primers used in real-time qPCR assays in this study are listed 5’-3’. F, forward; R, reverse; m, murine.

| Gene | Primer sequence | Gene | Primer sequence |
| --- | --- | --- | --- |
| *CHRM4* F | CTCCATGAACCTCTACACCGTG | *CHRM4* R | GACGCAGAAGTAGCGGTCAAAG |
| *CHGA* F | ACTGAAGGAGCTCCAAGAC | *CHGA* R | TCTGCCTCCTTGGAATCCTC |
| *ENO2* F | TCAGGGACTACCTGTGGTCT | *ENO2* R | TTCCACTGCCGCTCAATAC |
| *SYP* F | GGCTTTGTGAAGGTGCTGC | *SYP* R | CACTCTCGGTCTTGTTGGCA |
| *AR F* | AAGCAGTATCCGAAGGCAGC | *AR R* | AGAAATGGTCGAAGTGCCCC |
| *MYCN* F | ACAGTCATCTGTCTGGACGC | *MYCN* R | TGTCCTCGGATGGCTACAGT |
| *INHBC F* | CTGTGTCCAGAGCTGCTTTGAG | *INHBC R* | GACGAGTCTGGTTGATGGTGG |
| *PDL1* F | GGTAAGACCACCACCACCAA | *PDL1* R | TTGGAGGATGTGCCAGAGG |
| *CTLA4* F | ACGGGACTCTACATCTGCAAGG | *CTLA4* R | GGAGGAAGTCAGAATCTGGGCA |
| *IL1NR* F | GGAGGGAAGATGTGCCTGTC | *IL1RN* R | GTCCTGCTTTCTGTTCTCGCTC |
| *IFNG F* | GAGTGTGGAGACCATCAAGGA | *IFNG R* | TGCTTTGCGTTGGACATTCAAGT |
| *IFNA17 F* | GAAGACTCAAGCCATCTCTGTC | *IFNA17 R* | GGAGGCTCTGTTCCCAAGC |
| *18S* F | CATGGCCGTTCTTAGTTGGTGG | *18S* R | CGCTGAGCCAGTCAGTGTAG |

**Supplementary Table S3.** **Western blotting antibodies.** The source and dilution of each antibody used for Western blotting in this study are listed.

| Primary antibody | Clonality | Source  (Cat. No.) | Dilution | Secondary antibody | Source | Dilution |
| --- | --- | --- | --- | --- | --- | --- |
| CHRM4 | Polyclonal | sigma (SAB4300824) | 1:1000 | anti-rabbit IgG | Jackson Labs | 1:5000 |
| ENO2 | Monoclonal | Santa Cruz  (sc-21738) | 1:100 | anti-mouse IgG | Jackson Labs | 1:5000 |
| CHGA | Monoclonal | Santa Cruz  (sc-393941 ) | 1:100 | anti-mouse IgG | Jackson Labs | 1:5000 |
| AR | Monoclonal | Abcam (ab108341) | 1:1000 | anti-rabbit IgG | Jackson Labs | 1:5000 |
| KLK3 | Monoclonal | Santa Cruz  (sc-7316) | 1:200 | anti-mouse IgG | Jackson Labs | 1:5000 |
| NKX3-1 | Monoclonal | Thermo-Fisher (MA5-15618) | 1:2000 | anti-mouse IgG | Jackson Labs | 1:5000 |
| IFNA17 | Polyclonal | Abnova (H00003451-B01P) | 1:1000 | anti-mouse IgG | Jackson Labs | 1:5000 |
| PDL1 | Monoclonal | Proteintech (66248-1-lg) | 1:1000 | anti-mouse IgG | Jackson Labs | 1:5000 |
| CTLA4 | Monoclonal | cell signaling (#26893) | 1:1000 | anti-mouse IgG | Jackson Labs | 1:5000 |
| β-actin | Polyclonal | GeneTex (GTX109639) | 1:1000 | anti-rabbit IgG | Jackson Labs | 1:20000 |

**Supplementary Table S4.** **ChIP antibodies and primer sequences.** The source and dilution of each antibody and the sequences (5’-3’) of each primer used for ChIP in this study are listed.

| ChIP antibodies | | | | |
| --- | --- | --- | --- | --- |
| Primary antibody | Species | Clonality | Source | Dilution |
| AR | Rabbit | Monoclonal | Abcam (ab108341) | 1:50 |
| MYCN | Mouse | Monoclonal | Santa Cruz (sc-53993) | 1:50 |
| Acety-H3 | Rabbit | Monoclonal | Novus (NB300-221) | 1:100 |
| IgG | Rabbit |  | Santa Cruz (sc-2027) | 1:50 |
| ChIP primers | | | | |
| Site | Sequence | | | |
| *CHRM4*/ARE1 F | GCCCCGTGGCTGATAAGGAC | | | |
| *CHRM4*/ARE1 R | GCCTCTGTGGTGGACAGCTC | | | |
| *CHRM4*/ARE2 F | GGACCCCAAGAGAGGGCAGT | | | |
| *CHRM4*/ARE2 R | ACAGACCCAGCCTCCTAGCC | | | |
| *IFNA17*/EBOX1 F | CTGTGCTGGGGGTCTGCTAC | | | |
| *IFNA17*/EBOX1 R | GATGGCGCTCCCAAAGGGAA | | | |
| *IFNA17*/EBOX2 F | CGTCCTGCTTGGCTGGAGTT | | | |
| *IFNA17*/EBOX2 R | ACATGCAGGGCTACGTAGGGT | | | |
| *IFNA17*/EBOX3 F | GGATGGCAGCCAGTTGCAGA | | | |
| *IFNA17*/EBOX3 R | GAGCTGGTCCAGGAGGGTCA | | | |
| *PDL1*/EBOX1 F | TGGTGGGCTTCTGGATGGGA | | | |
| *PDL1*/EBOX1 R | TCTTCAGCCCCTCTCCCCTT | | | |
| *PDL1*/EBOX2 F | TCCGCCGATTTCACCGAAGG | | | |
| *PDL1*/EBOX2 R | GGGAAGCTGCGCAGAACTGG | | | |
| *PDL1*/EBOX3 F | GACACGGGTCCAAGTCCACC | | | |
| *PDL1*/EBOX3 R | GGACCTGCTTAGCGCAGGAA | | | |

**Reference**

1. Taylor BS, Schultz N, Hieronymus H, Gopalan A, Xiao Y, Carver BS*, et al.* Integrative genomic profiling of human prostate cancer. *Cancer Cell* 2010, **18**(1)**:** 11-22.

2. The Molecular Taxonomy of Primary Prostate Cancer. *Cell* 2015, **163**(4)**:** 1011-1025.

3. Beltran H, Prandi D, Mosquera JM, Benelli M, Puca L, Cyrta J*, et al.* Divergent clonal evolution of castration-resistant neuroendocrine prostate cancer. *Nat Med* 2016, **22**(3)**:** 298-305.

4. Wang G, Jones SJ, Marra MA, Sadar MD. Identification of genes targeted by the androgen and PKA signaling pathways in prostate cancer cells. *Oncogene* 2006, **25**(55)**:** 7311-7323.

5. Nelson PS, Clegg N, Arnold H, Ferguson C, Bonham M, White J*, et al.* The program of androgen-responsive genes in neoplastic prostate epithelium. *Proc Natl Acad Sci U S A* 2002, **99**(18)**:** 11890-11895.

6. Subramanian A, Tamayo P, Mootha VK, Mukherjee S, Ebert BL, Gillette MA*, et al.* Gene set enrichment analysis: a knowledge-based approach for interpreting genome-wide expression profiles. *Proc Natl Acad Sci U S A* 2005, **102**(43)**:** 15545-15550.

7. Yang JM, Chen CC. GEMDOCK: a generic evolutionary method for molecular docking. *Proteins* 2004, **55**(2)**:** 288-304.

8. Waterhouse A, Bertoni M, Bienert S, Studer G, Tauriello G, Gumienny R*, et al.* SWISS-MODEL: homology modelling of protein structures and complexes. *Nucleic Acids Res* 2018, **46**(W1)**:** W296-w303.

9. Thal DM, Sun B, Feng D, Nawaratne V, Leach K, Felder CC*, et al.* Crystal structures of the M1 and M4 muscarinic acetylcholine receptors. *Nature* 2016, **531**(7594)**:** 335-340.

10. Sterling T, Irwin JJ. ZINC 15--Ligand Discovery for Everyone. *J Chem Inf Model* 2015, **55**(11)**:** 2324-2337.
