# Supplementary figures and images for: CHRM4/AKT/MYCN upregulates interferon alpha-17 in the tumor microenvironment to promote neuroendocrine differentiation of prostate cancer
Source: Cell Death Dis. 2023 May 4;14(5):304. doi: 10.1038/s41419-023-05836-7 (PMC10160040; doi:10.1038/s41419-023-05836-7)

Fig. 1A

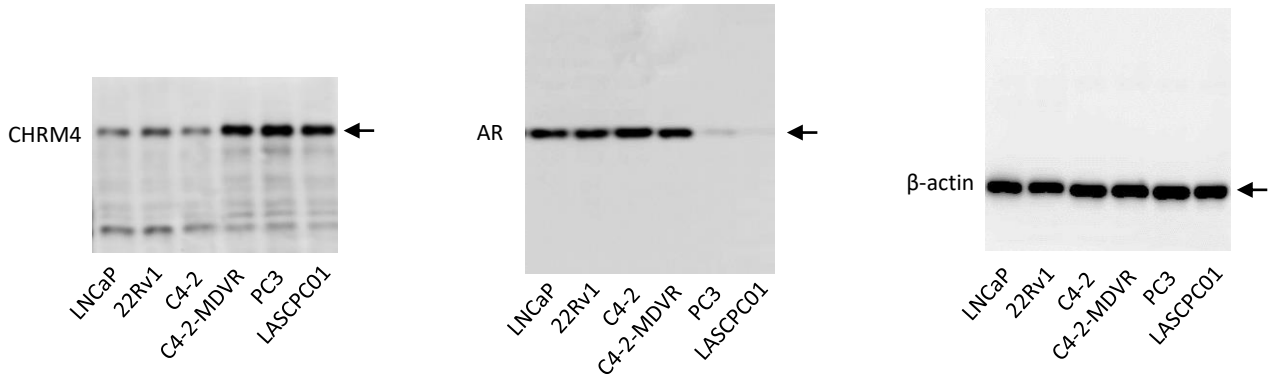

Fig. 1E

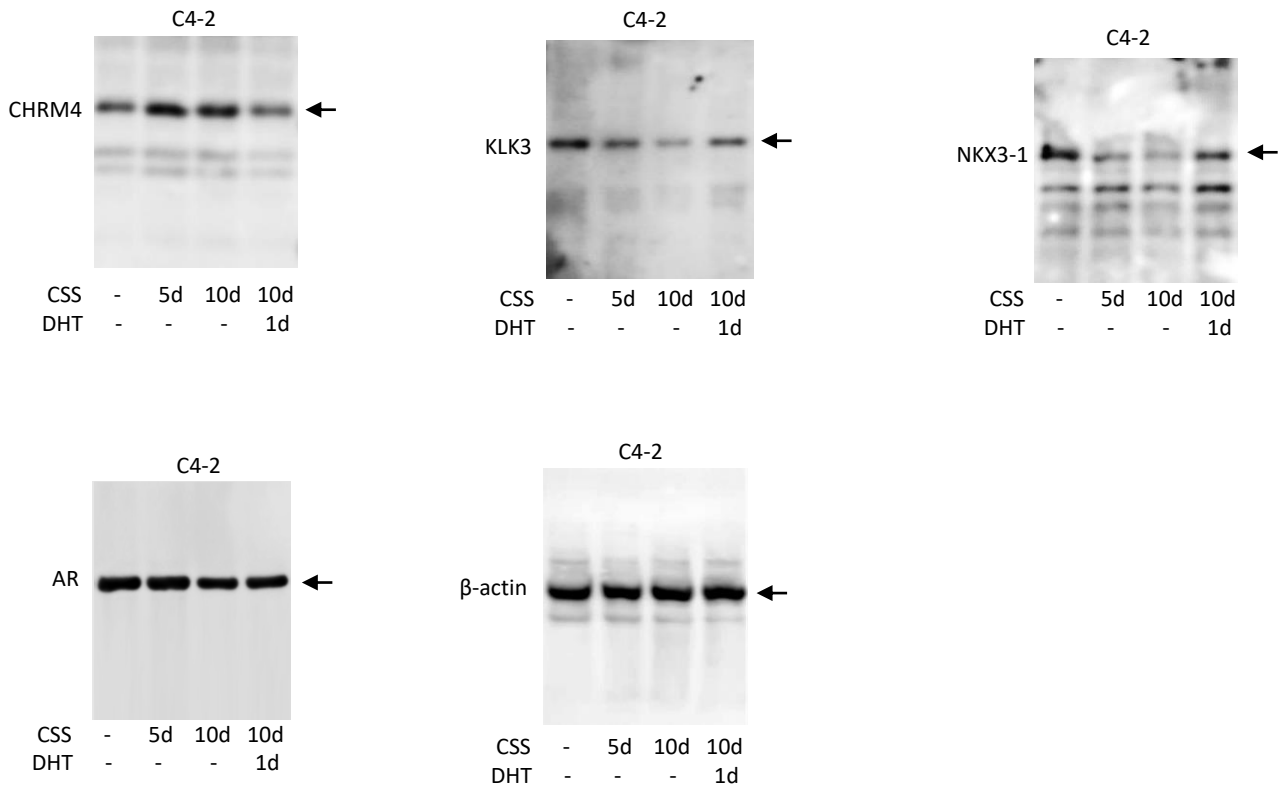

Fig. 1F

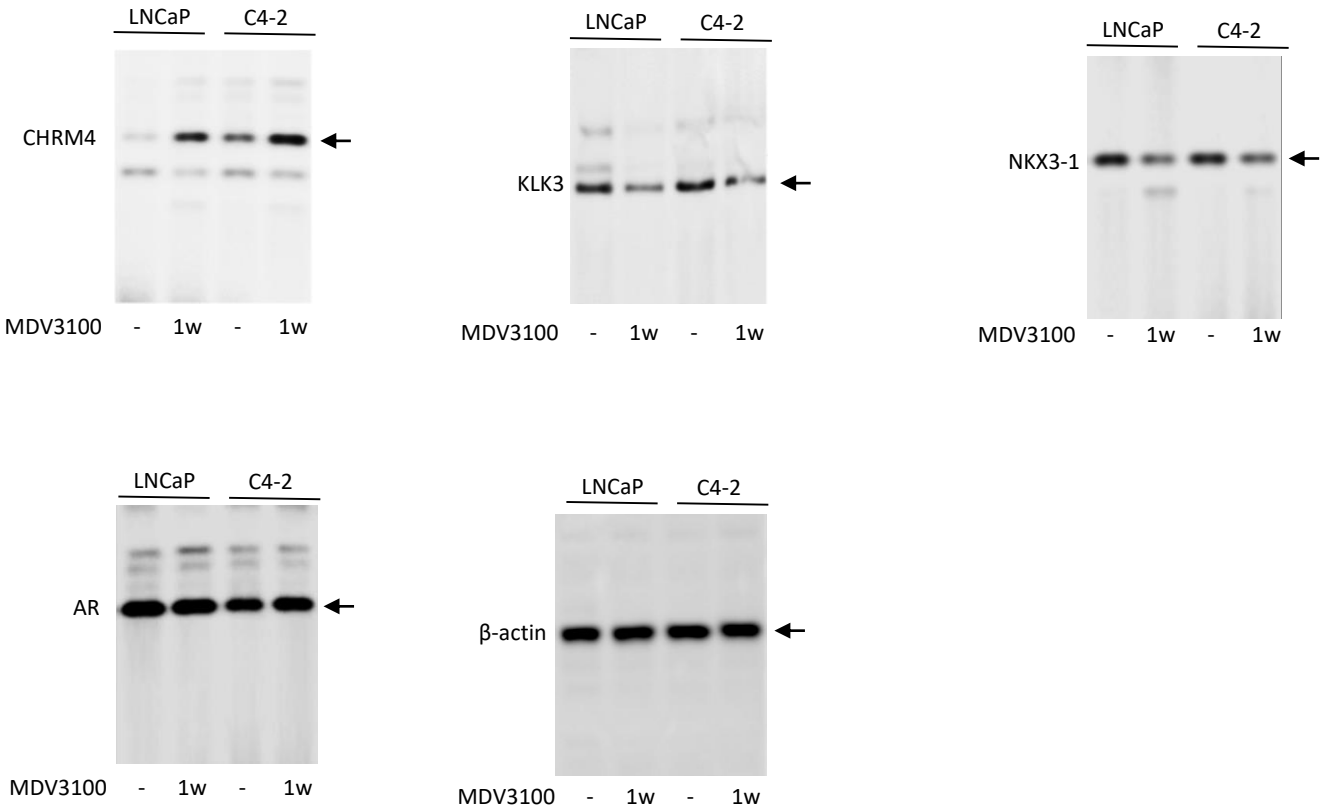

Fig. 3C

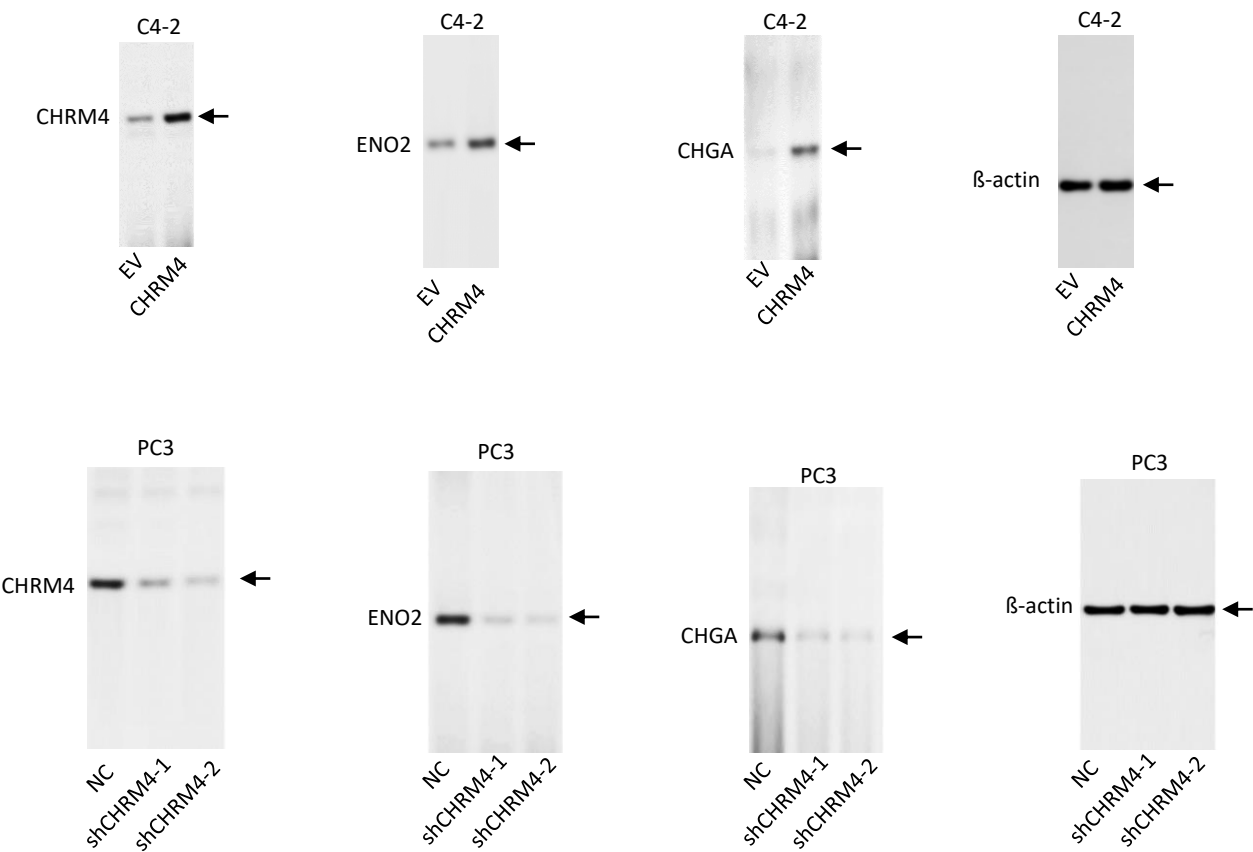

Fig. 4I

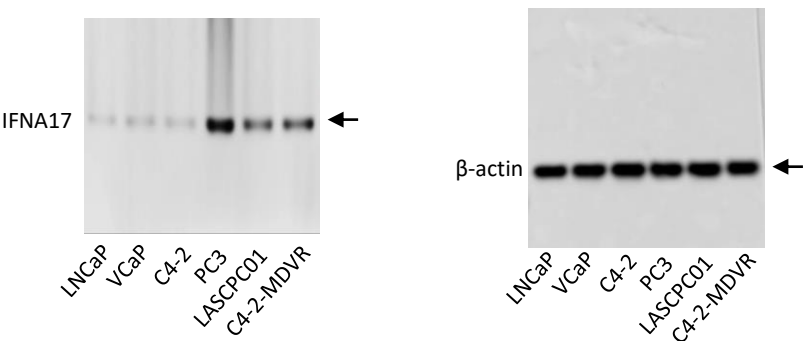

Fig. 5D

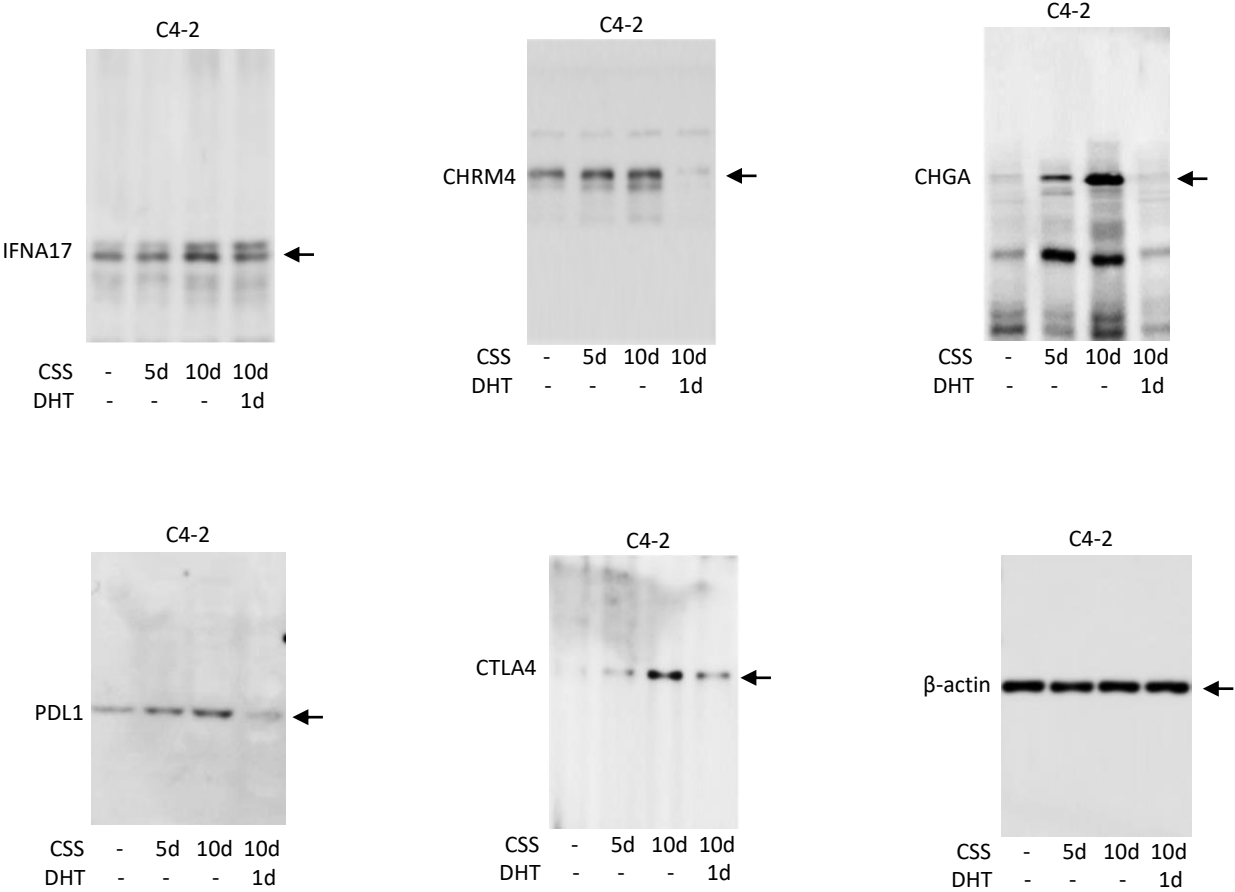

Fig. 5F

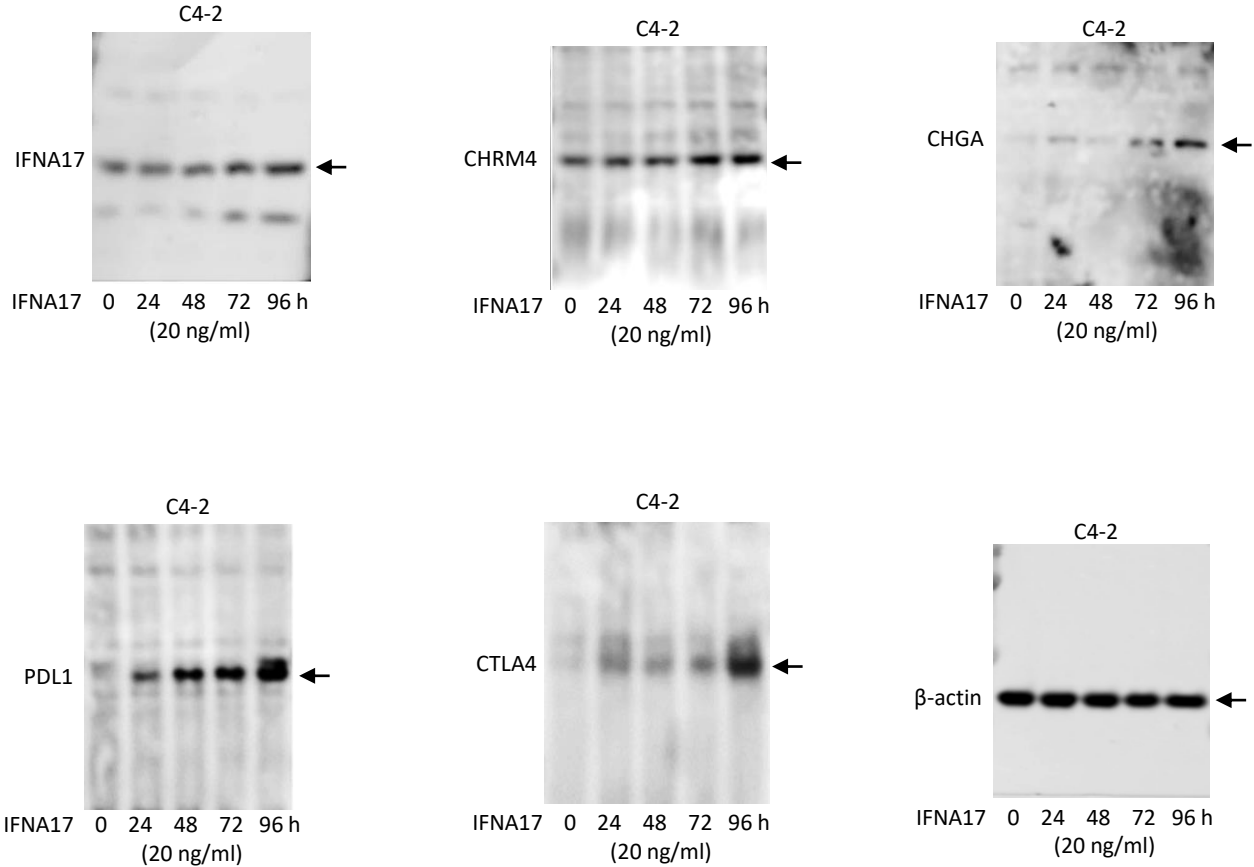

Fig. 5G

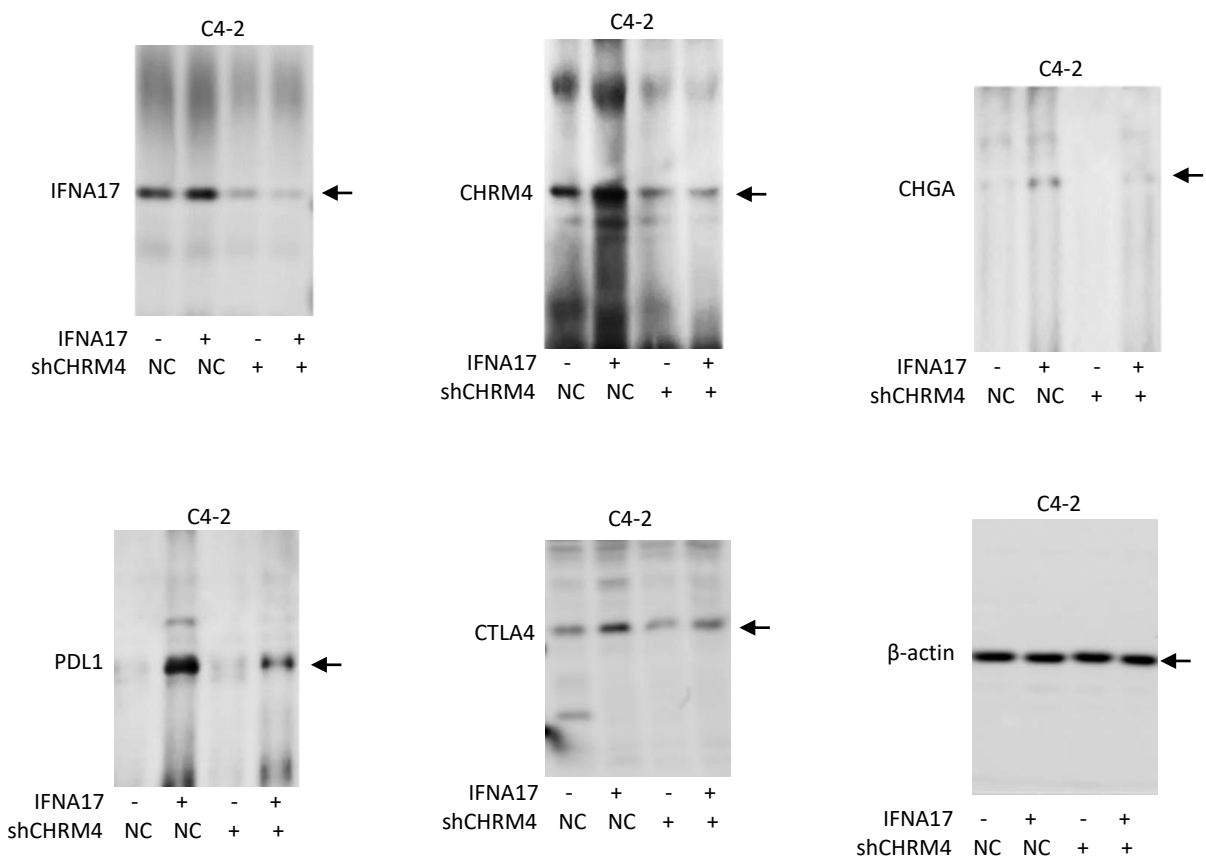

Fig. 5L

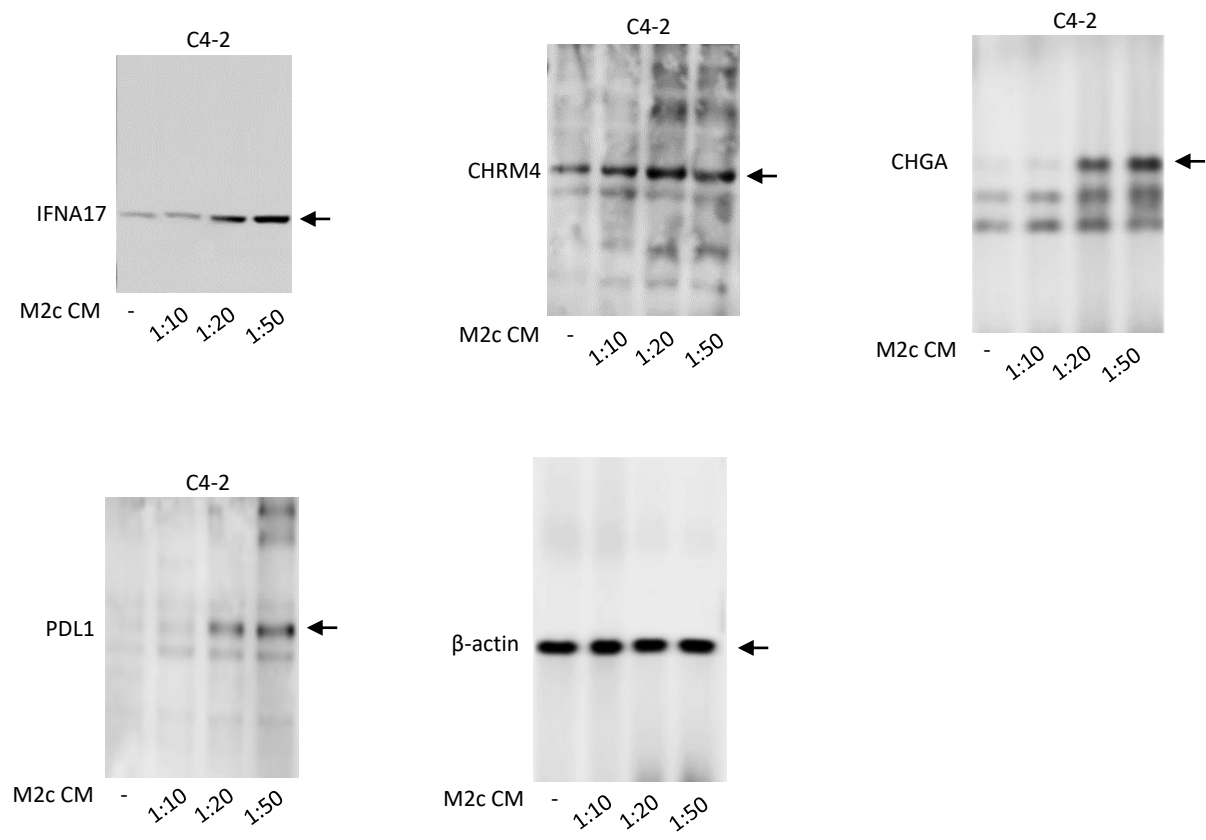

Fig. 6A

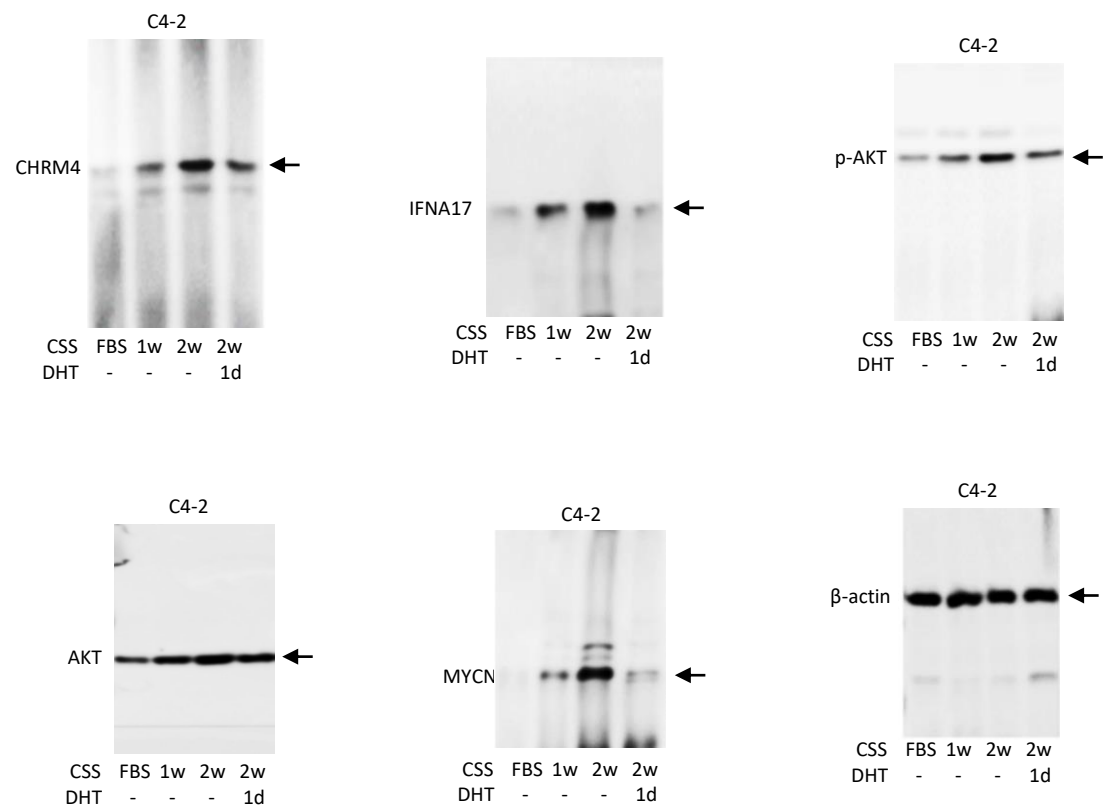

Fig. 6B

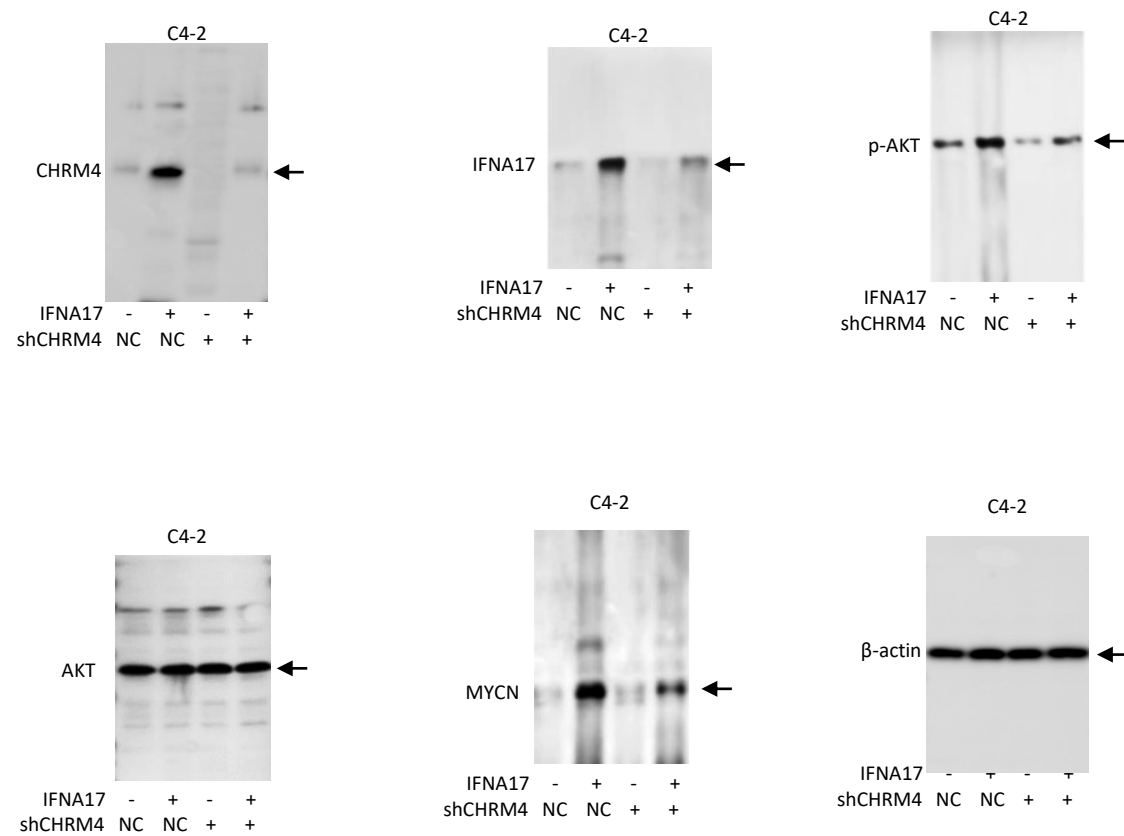

Supplement: Supplementary file 2 — Supplemental File 2 [file 41419_2023_5836_MOESM2_ESM.pdf]
